# (Electro‐)chemical Splitting of Dinitrogen with a Rhenium Pincer Complex

**DOI:** 10.1002/ejic.201901278

**Published:** 2020-01-31

**Authors:** Richt S. van Alten, Florian Wätjen, Serhiy Demeshko, Alexander J. M. Miller, Christian Würtele, Inke Siewert, Sven Schneider

**Affiliations:** ^1^ Institute of Inorganic Chemistry University of Goettingen Tammannstraße 4 37077 Goettingen Germany; ^2^ Department of Chemistry University of North Carolina at Chapel Hill 27599‐3290 Chapel Hill NC USA; ^3^ International Center for Advanced Studies of Energy Conversion University of Goettingen Tammannstraße 6 37077 Goettingen Germany

**Keywords:** Nitrogen fixation, Rhenium, Pincer complexes, Electrochemistry, Cyclic voltammetry

## Abstract

The splitting of N_2_ into well‐defined terminal nitride complexes is a key reaction for nitrogen fixation at ambient conditions. In continuation of our previous work on rhenium pincer mediated N_2_ splitting, nitrogen activation and cleavage upon (electro)chemical reduction of [ReCl_2_(*L*2)] {*L*2 = N(CHCHP*t*Bu_2_)_2_^–^} is reported. The electrochemical characterization of [ReCl_2_(*L*2)] and comparison with our previously reported platform [ReCl_2_(*L*1)] {*L*1 = N(CH_2_CH_2_P*t*Bu_2_)_2_^–^} provides mechanistic insight to rationalize the dependence of nitride yield on the reductant. Furthermore, the reactivity of N_2_ derived nitride complex [Re(N)Cl(*L*2)] with electrophiles is presented.

## Graphical abstract

N_2_ splitting into terminal nitrides by chemical and electrochemical reduction of [ReCl_2_{N(CHCHP*t*Bu_2_)_2_}] is presented. Comparison of electrochemical data with that of the previously reported, related pincer complex [ReCl_2_{N(CH_2_CH_2_P*t*Bu_2_)_2_}] allowed for identifying key parameters that control the efficiency of the reaction sequence, which defines reductive N_2_ splitting.

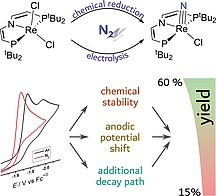

## Introduction

Industrial ammonia synthesis by the Haber–Bosch process is carried out at a scale of 150 Mt/a, using hydrogen produced via steam reforming of fossil fuels that accounts for massive energy consumption and CO_2_ emission.^1^ The replacement of H_2_ as reductant is therefore highly desirable to enhance the sustainability of nitrogen fixation. The electrochemically driven nitrogen reduction reaction (NRR) is an appealing alternative to feed renewable energy from photovoltaic harvesting.^2^ Electrocatalytic NRR has seen tremendous progress in recent years. Faradaic yields up to 73.3 % have been reported, yet with current densities far below the US Department of Energy targets.^3^, ^4^ Furthermore, the mechanistic basis of heterogeneous electrocatalysts remains comparatively ill‐defined. Homogeneous (model) NRR catalysts could give detailed insight on key reaction steps and thermochemical and kinetic parameters.^5^ However, molecular NRR electrocatalysts are highly limited.^6^

**Scheme 1:**
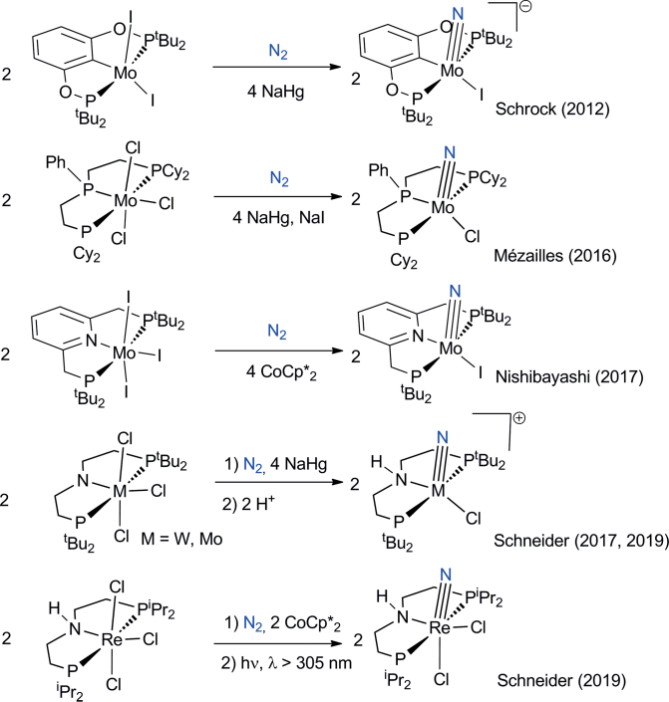
Selected examples for N_2_‐splitting into terminal nitride complexes with transition metal pincer platforms.

**Scheme 2:**
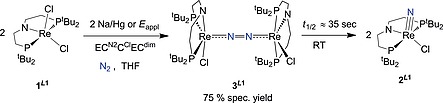
(Electro)chemical N_2_‐activation from [ReCl_2_(*L*1)] (**1^*L*1^**) into [Re(N)Cl(*L*1)] (**2^*L*1^**) via the N_2_‐bound dimeric intermediate [{ReCl(*L*1)}_2_(µ‐N_2_)] (**3^*L*1^**) formed via an *EC* ^N2^ *C* ^Cl^ *EC* ^dim^ type mechanism.

Two general mechanisms have been proposed for the NRR with molecular catalysts. The “bio‐inspired” route is comprised of successive proton coupled electron transfer (PCET) steps at terminally coordinated N_2_, in analogy to the mechanism proposed for the [Fe,Mo]‐nitrogenase enzyme.^7^, ^8^ Initial full cleavage of the N≡N triple bond via N_2_‐bridged, multinuclear complexes and subsequent PCET of the resulting nitrides, as in the Fe‐catalyzed Haber–Bosch process, has been alternatively considered.^9^ The splitting of dinitrogen into well‐defined nitride complexes was pioneered by Laplaza and Cummins 25 years ago and several examples are known by now.^10^, ^11^ Recently, group 6 and 7 pincer platforms attracted particular attention (Scheme 1).^9^, ^12^ Our group reported N_2_ splitting upon chemical reduction [Na/Hg, Co(Cp*)_2_] of the rhenium(III) PNP pincer complex [ReCl_2_(*L*1)] {(**1^*L*1^**; *L*1 = N(CH_2_CH_2_P*t*Bu_2_)_2_)^–^} to the nitrido complex [Re(N)Cl(*L*1)] (**2^*L*1^**; Scheme 2).[12b] Miller, Siewert, Schneider and co‐workers jointly examined electrochemically driven N_2_ cleavage for this platform, which allowed for detailed mechanistic study by cyclic voltammetry (CV).[12g] The reaction goes through rate determining splitting of the N_2_‐bridged dirhenium complex [{ReCl(*L*1)}_2_(µ‐N_2_)] (**3^*L*1^**; *t*_1/2_^298K^ ≈ 35 s). Intermediate **3^*L*1^** is formed within a complex *EC* ^N2^ *C* ^C^ *^l^EC* ^dim^ pathway via (electro)chemical Re^III^/Re^II^ reduction (*E*_1_) of **1^*L*1^**, followed by N_2_ binding (*C* ^N2^), chloride loss (*C* ^Cl^), Re^II^/Re^I^ reduction (*E*_2_) and subsequent comproportionation with parent **1^*L*1^** (*C* ^dim^). Besides mechanistic insight, this study provided the first example of N_2_ splitting into nitrido complexes by controlled potential electrolysis (CPE at –1.90 V vs. Fc^+/0^) with yields around 60 %. Recently, Masuda and co‐workers demonstrated electrochemically driven N_2_ splitting upon anodic oxidation of *trans*‐[Mo(N_2_)_2_(depe)_2_] (depe = Et_2_PCH_2_CH_2_PEt_2_).^13^ However, further systematic studies are required to identify the key parameters that control the N_2_ splitting reaction.

Here, (electro‐)chemical N_2_ splitting with a modified Re pincer platform is reported. The divinylamide ligand N(CHCHP*t*Bu_2_)_2_^–^ (*L*2^–^) was previously utilized for the stabilization of a wide variety of transition metal complexes.^14^, ^15^ The enhanced rigidity resembles that of “archetypical” amide pincer ligands, like Milstein's pyridine‐based dearomatized ligand NC_5_H_3_(2‐CHP*t*Bu_2_)(6‐CHP*t*Bu_2_)^–^, with increased steric protection, as compared to phenylene‐bridged diphosphinoamide N(C_6_H_4_P*i*Pr_2_)_2_^–^.^16^ In comparison to parent *L*1, backbone unsaturation leads to significant reduction of N→M π‐donation, as reflected in CO and N_2_ stretching frequencies of Ru and Ir complex series.^14^, [15e], [15k] Starting from [ReCl_2_(*L*2)] (**1^*L*2^**), the effects of backbone unsaturation on the reduction potential, N_2_ splitting yields and functionalization of the nitride product are discussed.

## Results and Discussion

### Synthesis and Characterization of 1^*L*2^

**Scheme 3:**
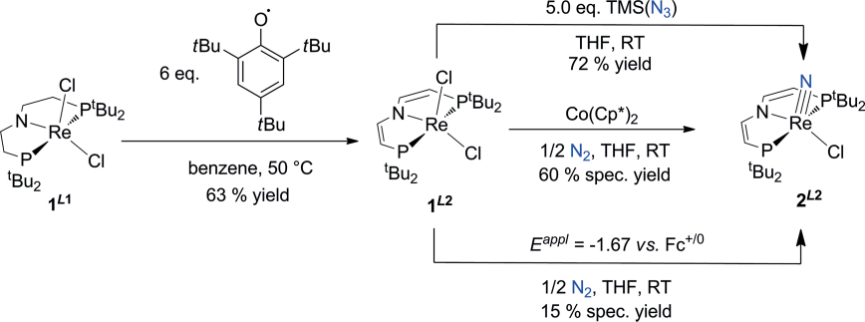
Synthesis of complex **1^*L*2^** by ligand oxidation starting from **1^*L*1^** using the 2,4,6‐*tert*‐butylphenoxy radical and different routes for the synthesis of complex **2^*L*2^** by either (electro)chemical N_2_‐splitting, or via reaction with TMS(N_3_).

Complex **1^*L*2^** was synthesized starting from **1^*L*1^** by templated ligand modification via hydrogen atom abstraction with excess 2,4,6‐*tert*‐butylphenoxy radical (TBP) at 50 °C (Scheme 3), as similarly reported for other *L*2 complexes.^15^ Small amounts of a paramagnetic side‐product found by ^1^H NMR spectroscopy could be identified as overoxidized rhenium(IV) complex [ReCl_3_(*L*2)] (**4^*L*2^**) upon comparison with an original sample that was independently synthesized. Facile conversion of the side product **4^*L*2^** to **1^*L*2^** is accomplished by in situ reduction with Co(Cp)_2_, providing the analytically pure product in 63 % isolated yield. The ^1^H NMR spectrum of **1^*L*2^** indicates *C*_2*v*_ symmetry in solution. In the ^31^P{^1^H} NMR spectrum, a sharp singlet resonance was found at *δ*_31P_ = –275 ppm (Figure S3). In analogy to other rhenium(III) phosphine complexes and **1^*L*1^**,[12h], 17 the high‐field shift is attributed to mixing of the ground‐state with low‐lying excited states leading to temperature independent paramagnetism (TIP),^19^ as substantiated for **1^*L*1^** and **1^*L*2^** by SQUID magnetometry {*χ_M_*[10^–6^ × cm^3^ mol^–1^] = 280 (**1^*L*1^**), 300 (**1^*L*2^**); Figure S26}. Despite several attempts, single crystals of **1^*L*2^** suitable for X‐ray analysis could not be obtained.

### N_2_ Splitting by (Electro‐)Chemical Reduction

Reduction of **1^*L*2^** with an equimolar amount of Co(Cp*)_2_ in THF under 1 atm N_2_ results in rapid conversion to a mixture of several diamagnetic products, according to ^1^H and ^31^P{^1^H} NMR spectroscopy. The rhenium(V) nitride [Re(N)Cl(*L*2)] (**2^*L*2^**) was identified as the major species (60 % yield by NMR spectroscopy, see Figure S7) by comparison to an authentic sample prepared by reaction of **1^*L*2^** with trimethylsilyl azide (Scheme 3). All attempts to identify intermediates by NMR monitoring at low temperatures were unsuccessful. The yield in **2^*L*2^** is slightly lower compared with parent **2^*L*1^** [75 % with Co(Cp*)_2_] and notably depends on the reductant. Considerably lower spectroscopic nitride yields are obtained with alkali metal reductants, such as Na/Hg (approx. 30 %) or KC_8_ (approx. 20 %), under otherwise identical conditions. In comparison, 80 % yield in **2^*L*1^** was obtained upon reducing **1^*L*1^** with Na/Hg under N_2_. Notably, with Na/Hg or KC_8_ as reductant, yet not with Co(Cp*)_2_, the liberation of isobutene was detected spectroscopically for **1^*L2*^** (Figure S14), as previously observed for the thermal decomposition of [OsCl(*L*2)],[15j] suggesting fragmentation of the *L*2 ligand platform upon overreduction. Strong dependence of N_2_ splitting yields on the nature of the reductant has been previously reported.^21^ However, in most cases these effects are poorly understood.

**Figure 1:**
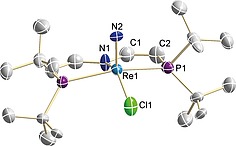
Molecular structure of **2^*L*2^** from single‐crystal X‐ray diffraction with anisotropic displacement parameters drawn at the 50 % probability level. Hydrogen atoms are omitted for clarity. Selected bond lengths [Å] and angles [°]: Re1–N1 2.106(3), Re1–Cl1 2.395(7), Re1–N2 1.647(18), Re1–P1 2.447(3), C1–C2 1.35(2), N1–Re1–Cl1 145.9(2), N1–Re1–N2 109.2(5), Cl1–Re1–N2 104.8(6), P1–Re–P2 155.1(1).

Multinuclear NMR spectroscopic characterization of **2^*L*2^** indicates *C*_s_ symmetry with a peak at *δ*_31P_ = 71.8 ppm in the ^31^P{^1^H} NMR spectrum. Single crystal X‐ray characterization (Figure 1) reveals a slightly distorted square pyramidal geometry (*τ*_5_ = 0.15)^22^ with the nitride [Re–N2 1.647(18) Å] in apical position. These bond metrics are close to those of **2^*L1*^** [Re≡N = 1.643(6) Å, *τ*_5_ = 0.14], which was recently characterized crystallographically.^23^ The planar ligand backbone with shortened C=C bonds [**2^*L*2^**: 1.35(2) Å; **2^*L*1^**: 1.545(10)/1.526(10) Å] confirms the presence of vinylene linkers in the pincer ligand backbone. Electrochemical characterization of the nitrido species was carried out by cyclic voltammetry (CV) in THF (Figure S21). A reversible oxidation at +0.21 V (vs. Fc^+/0^)^24^ was assigned to the Re^V^/Re^VI^ couple and is significantly anodically shifted with respect to **2^*L*1^** (*E*_1/2_ = –0.086 V).[12g] This potential shift is consistent with reduced electron density at the rhenium ion of **2^*L*2^** due to weaker donation by pincer ligand *L*2. **2^*L*2^** features an additional, irreversible reduction feature at low potential (*E*_p,c_ = –3.3 V vs. Fc^+/0^).

**Figure 2:**
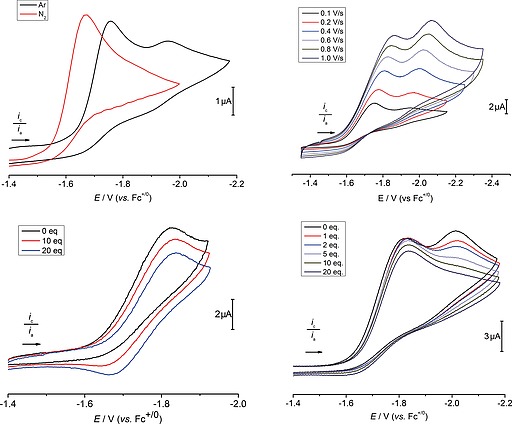
CVs of **1^*L*2^**. *Top Left*: Ar (black) and N_2_ (red) at *ν* = 0.1 V s^–1^. *Top Right*: Scan rate dependence under Ar. *Bottom Left*: Under Ar, in the presence of varying amounts of (*n*Bu_4_N)Cl (*v* = 0.5 V s^–1^). *Bottom Right*: Under Ar, in the presence of varying amounts of (*n*Bu_4_N)Cl (*v* = 0.5 V s^–1^). General conditions: 1.0 mm **1^*L*2^** in THF, 0.2 m (*n*Bu_4_N)PF_6_.

CPE of **1^*L*2^** under 1 atm N_2_ was carried out in THF at *E* = –1.67 V, i.e. the cathodic peak potential of the first reductive feature (Figure 2, top left; vide infra for discussion). Thus, the use of ligand *L*2 enables electrolysis at approx. 230 mV less negative potential with respect to **1^*L*1^**, presumably due to the poorer π‐donor properties of the unsaturated pincer. Transfer of approximately 1.2 electrons per Re over the course of 2 h was accompanied by a gradual color change from brown to light brown/green. Spectroscopic yields of nitride **2^*L*2^** of approx. 15 % were obtained (Figure S8), which are significantly lower than the electrolysis yields of nitride **2^*L*1^** (60 %). The low electrolysis yield in **2^*L*2^** is in stark contrast with Co(Cp*)_2_, and closer to other heterogeneous reductants (Na/K, KC_8_).

**Scheme 4:**
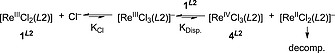
Proposed chloride‐induced disproportionation of **1^*L*2^**.

In order to rationalize the lower N_2_ splitting yields, the stability of **1^*L*2^** in THF in the presence of N_2_ and chloride ions was assessed. NMR spectroscopic monitoring under 1 atm N_2_ reveals partial conversion to several unidentified new species in the spectral range *δ*_31P_ = 20–60 ppm (Figure S15). CV characterization at higher N_2_‐pressure initially shows a slight rise in the current of the reduction feature by around 5 % upon increasing pressure from 1 to up to 11 bars (Figure S20). However, over the course of 45 min at 11 bars of N_2_ (see Experimental Section), the current drops by about 20 % suggesting chemical instability of **1^*L*2^** under these conditions. ^31^P{^1^H} NMR spectroscopic analysis after this experiment shows complete conversion of **1^*L*2^** to an intractable reaction mixture (Figure S16). More rapid decay was even found upon addition of a chloride source, suggesting that accumulation of chloride ions released during electrolysis may accelerate decomposition. A mixture of **1^*L*2^** and (*n*Bu_4_N)Cl under 1 atm N_2_ gradually changes color from light brown to green over the course of a couple of hours, with concomitant formation of a mixture of diamagnetic and paramagnetic species (Figure S17). Comparison with ^1^H NMR spectra of mixtures of authentic **1^*L*2^**, (*n*Bu_4_N)Cl, and **4^*L*2^** (Figure S18) supports the assignment of a broad signal at +12 ppm to rhenium(IV) complex **4^*L*2^**. This observation suggests that the chloride‐induced decay of **1^*L*2^** proceeds via disproportionation of [Re^III^Cl_3_(*L*2)]^–^ to **4^*L*2^** and further, unstable rhenium(II) species as outlined in Scheme 4.

The relevance of the decay pathway shown in Scheme 4 for the electrochemical transformations was evaluated from available thermochemical data (see also Electronic Supporting Information, Section 5). The invariance of *δ*_31P_(**1^*L*2^**) and inability to detect a new signal for [ReCl_3_(*L*2)]^–^ in the presence of added chloride (5 equiv.) allows for estimating an upper limit of the chloride association constant (*K*_Cl_ ≤ 0.015 m ^–1^; Δ*G* ^0^_Cl_ ≥ +2.5 kcal mol^–1^). Subsequent disproportionation of [Re^III^Cl_3_(*L*2)]^–^ with **1^*L*2^** to [Re^II^Cl_3_(*L*2)]^–^ and **4^*L*2^** is defined by the reduction potentials of **4^*L*2^** (*E*_1/2_ ≈ –0.9 V vs. Fc^+/0^; Figure S22) and **1^*L*2^** (*E*_1/2_ = –1.75 V vs. Fc^+/0^; vide infra), giving *K*_Disp_ ≈ 4 × 10^–15^ and Δ*G* ^0^_Disp_ ≈ +20 kcal mol^–1^. The chloride‐induced decomposition pathway outlined in Scheme 4 would therefore have to be driven by the decay of [ReCl_2_(*L*2)]^–^. However, the overall effective kinetic barrier needs to be larger than Δ*G* ^‡^ ≥ 22.5 kcal mol^–1^. In consequence, chloride induced decomposition is irrelevant on the CV timescale but might reduce electrolysis yields, which goes over hours.

In comparison, parent **1^*L*1^** proved stable under these conditions over an extended period of time. Structural comparison of **2^*L*1^** and **2^*L*2^** shows only minor differences, like the steric shielding as expressed by the pincer bite angle [P–Re–P: 156.16(7)° (**2^*L*1^**), 155.11(13)° (**2^*L*2^**)]. We therefore tentatively associate the reduced stability to electronic reasons. Backbone unsaturation changes the donor properties (poorer π‐donation) and increases the metal Lewis acidity. Furthermore, ligand *L*2 is potentially non‐innocent and can undergo versatile proton/electron transfer at the vinyl groups.[15i] The reduced stability of **1^L2^** in the presence of N_2_ and chloride will contribute to lowering the electrolysis yields. Electrochemical reduction occurs on a longer time scale (2 h) than chemical N_2_‐splitting, e.g. with Co(Cp*)_2_ as reductant (5 min). Thus, **1^*L*2^** will be exposed to N_2_ and released free chloride during electrolysis for a longer time. However, the estimated decay rates suggest that further processes contribute to the low nitride electrolysis yields. Therefore, the reduction of **1^*L*2^** was examined in depth by CV, which is presented in the next section.

### CV Examinations

The CV of **1^*L*2^** under Ar (Figure 2) reveals two irreversible, reductive features at *E*_p,c,1_ = –1.75 V and *E*_p,c,2_ = –1.95 V (vs. Fc^+/0^; *ν* = 0.1 V s^–1^), respectively. The peak currents *i*_p,c,1_ and *i*_p,c,2_ scale linearly with *v* ^1/2^, indicating diffusion‐controlled electron transfer. Both reductions exhibit distinct cathodic potential shifts with rising current ratio *i*_p,c,1_/*i*_p,c,2_ at increasing scan rates (Figure 2, *top right*). The current characteristics suggest the presence of competing chemical reaction pathways after initial reduction of **1^*L*2^** including decay to a redox‐inactive species.

Changing from Ar to N_2_ (1 bar), the irreversible first reduction of **1^*L*2^** shifts anodically by about 85 mV to *E*_p,c_ = –1.67 V (Figure 2, *top left*) accompanied by a small peak current increase (approx. 5 %). The second reduction feature present under Ar vanishes under N_2_ without appearance of new reductive events. The anodic potential shift and the disappearance of the Re^II^/Re^I^ reduction are in agreement with N_2_‐activation at the rhenium(II) stage, as proposed for **1^*L*1^**.[12g] The anodic shift with respect to **1^*L*1^** (approx. 230 mV) compares well with the shift found for the corresponding nitrides **2^*L*1^** and **2^*L*2^** (vide infra) and is therefore associated with weaker π‐donation by pincer ligand *L*2. Besides the first reduction (Re^III^/Re^II^), the second reduction feature (Re^II^/Re^I^) that is obtained in the absence of N_2_ is even more anodically shifted, leading to decreased peak separation for **1^*L*2^** (Δ*E* = 0.17 V) as compared to **1^*L*1^** (Δ*E* = 0.29 V). In consequence, strong reductants, like Na/Hg (*E*° < –2.3 V),^25^ have potentials that are well beyond the Re^II^/Re^I^ couple of **1^*L*2^**. Unproductive overreduction in case of incomplete trapping of the rhenium(II) intermediate by dinitrogen might therefore be a contributing factor to the lower nitride yields obtained with Na/Hg or KC_8_, respectively, vs. Co(Cp*)_2_ (*E*° = –1.84 V).^26^

Further insight was obtained by electrochemical evaluation at varying conditions. Due to the limited chemical stability of **1^*L*2^** in the presence of N_2_ and low electrolytic Faradaic yields, we focused on the decay kinetics under argon to identify pathways that could lead to the reduced nitride yields with respect to **1^*L*1^**. The effect of added (*n*Bu_4_N)Cl on the CV response was examined to probe for coupled chloride loss. Modest increase in reversibility and a slight cathodic shift are obtained for the first reduction event *E*_1_ with rising chloride concentrations (Figure 2, *bottom left*), in line with coupled, fast and reversible chloride dissociation after reduction of **1^*L*2^**. The peak current decrease is attributed to slow decomposition of **1^*L*2^** in presence of excess chloride (vide supra). Scanning both reduction events *E*_1_ and *E*_2_ (Figure 2, bottom right), the second feature drops in current and shifts cathodically with increasing chloride ion concentration. The concentration dependence in **1^*L*2^** (0.5–4.0 mm) shows increasing *i*_p,c,1_/*i*_p,c,2_ current ratio at higher *c*_Re_ (Figure S20), indicating a bimolecular decay route between the two reduction events.

**Scheme 5:**
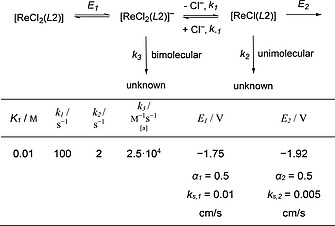
Minimum kinetic model for the digital simulation of the electrochemical reduction of **1^*L*2^** under Ar and thermodynamic and kinetic parameters (formal potentials, rate constants, and electron transfer parameters) obtained from CV data simulation; ^[a]^ for bimolecular decay of [ReCl_2_(*L*2)]^–^.

Our previous electrochemical study for the reduction of parent **1^*L*1^** allowed for rationalization of the CV data under Ar by an *EC* ^Cl^ *E* minimum model with Re^III^/Re^II^ and Re^II^/Re^I^ redox couples that are connected by chloride dissociation between electron transfers.[12g] Quantitative kinetic modelling by digital simulation of the CV data further required the introduction of a unimolecular decay step at the rhenium(II) stage *after* chloride loss. For **1^*L*2^**, the data indicates at least two coupled chemical reactions after the first reduction event: chloride dissociation that forms [ReCl(*L*2)] (as proposed for **1^*L*1^**) *and* competing bimolecular decay of [ReCl_2_(*L*2)]^–^, respectively. A best fit over all CV data of **1^*L*2^** under Ar was found for the kinetic model and simulation parameters presented in Scheme 5.

**Figure 3:**
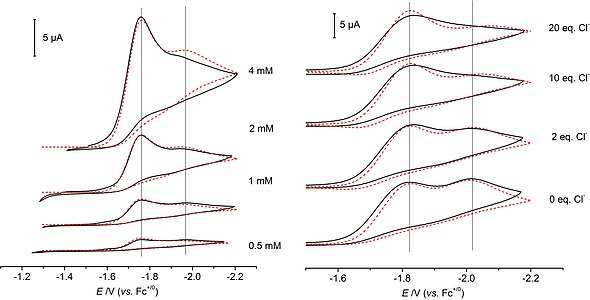
Experimental (black lines) and simulated (red dashed lines) CV data [0.2 m (*n*Bu_4_N)PF_6_ in THF] of **1^*L*2^** under Ar; mechanism and simulation parameters according to Scheme 5. *Left*: Concentration dependent data, *ν* = 0.1 V s^–1^. *Right*: Chloride dependent data, *ν* = 0.5 V s^–1^.

Typical simulation data are shown in Figure 3 and Figures S24/S25. Within the model, reduction of **1^*L*2^** (*E*_1_) is succeeded by reversible chloride dissociation (*K*_1_) and irreversible Re^II^/Re^I^ reduction (*E*_2_). Importantly, a satisfactory minimum model required two decay routes to account for the concentration dependence of *i*_p,c,1_/*i*_p,c,2_: unimolecular decay of [ReCl(*L*2)] (*k*_2_) after chloride loss as proposed for **1^*L*1^**, but also bimolecular decay before chloride dissociation (*k*_3_). Assuming formation of electrochemically silent species, bimolecular decay of [ReCl_2_(*L*2)]^–^ was modeled since an alternative reaction of [ReCl_2_(*L*2)]^–^ with parent [ReCl_2_(*L*2)] would exhibit decreasing normalized *i*_p,c,1_ at increasing concentration, which is not observed.

It is tempting to assume disproportionation of Re^II^ to Re^III^ and Re^I^ as bimolecular pathway. However, simple disproportionation, e.g., of [ReCl_2_(*L*2)]^–^ to [ReCl(*L*2)]^–^ and parent **1^*L*2^** after chloride loss from [ReCl_3_(*L*2)]^–^ should lead to increasing overall currents at higher chloride concentrations, which is not in agreement with the data. In consequence, disproportionation requires the introduction of additional decay routes, e.g. at the Re^I^ stage, which was renounced to avoid overparameterization of the model. However, disproportionation cannot be fully excluded.

The quality of the simulations is quite sensitive with respect to doubling or halving the decay rate constants *k*_2_ or *k*_3_, respectively. However, the two parameters are correlated: a higher bimolecular rate constant *k*_3_ could be partially compensated by lower *k*_2_ (and vice versa), yet with poorer resemblance of reversibility. For the rate and equilibrium constants of chloride loss (*k*_1_, *K*_1_), the fit proved highly sensitive with respect to variations.

Rapid N_2_‐activation (*k* > 5 × 10^7^ M^–1^s^–1^) by anionic [Re^II^Cl_2_(*L*1)]^–^ was demonstrated as key step for N_2_ splitting with **1^*L*1^**.[12g] Thus, the lifetime of the rhenium(II) intermediate predetermines the N_2_ splitting yield. In case of **1^*L*2^**, the chloride dissociation preequilibrium (*K*_1_) is followed by unimolecular decay (*k*_2_) that is about an order of magnitude faster as compared with **1^*L*1^**. In addition, a bimolecular decay pathway (*k*_3_) prior to chloride loss may further reduce the lifetime of rhenium(II) species. Besides lowering the electrosynthetic yield, the bimolecular decay may also be detrimental for heterogeneous reductants (Na/Hg, KC_8_). There, high local surface concentrations of reduced species are expected as opposed to homogeneous reduction, e.g. with Co(Cp*)_2_, which gave the highest N_2_ splitting yields for **1^*L*2^**.

### Nitride Functionalization

**Scheme 6:**
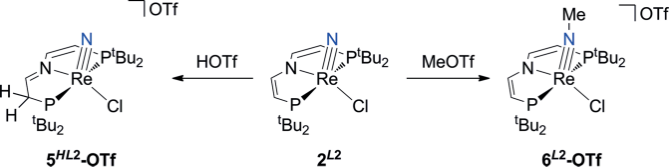
Reactivity of **2^*L*2^** towards electrophiles. Reaction with HOTf results in backbone protonation, whereas MeOTf leads to C–N bond formation by methylation at the nitride.

The functionalization of the nitride complex **2^*L*2^** derived from N_2_ splitting was investigated. No reactivity was found with ONMe_3_, PMe_3_, or CO, indicating that the weaker donor properties of the pincer ligand do not open up pathways for potential nucleophiles/ambiphiles. However, in analogy to **2^*L*1^**, **2^*L*2^** readily reacts with strong electrophiles, such as triflic acid and methyl triflate (Scheme 6). With triflic acid in Et_2_O, almost quantitative protonation of a vinyl group in the pincer backbone and formation of [Re(N)Cl(H*L*2)]OTf (**5^H*L*2^‐OTf**) is evidenced by the NMR signature, such as the two ^31^P{^1^H}‐NMR signals with typical *trans* coupling constant (^2^ *J*_PP_ = 148 Hz). The same reactivity of *L*2 complexes with Brønsted acids was previously found for nickel(II), cobalt(II), and ruthenium(II) complexes.[15h], [15i][15k] Electrochemical examination of **5^H*L*2^‐OTf** in THF revealed a reversible oxidation at *E*_1/2_ = +0.24 V (Figure S23), yet no reductive process within the potential window of THF.

In contrast to protonation, treatment of **2^*L*2^** with MeOTf in chlorobenzene at elevated temperatures results in functionalization of the N_2_ derived nitride group (Scheme 6). The imido complex [Re(NMe)Cl(*L*2)]OTf (**6^*L*2^‐OTf**) with a single ^31^P{^1^H} signal at *δ* = 88.8 ppm is obtained. Nitride methylation by the electrophile was confirmed by ^1^H‐^1^H NOESY spectroscopy, which shows cross‐peaks of the N–C*H*_3_ group at *δ* = 2.70 pm with one of the two *tert*‐butyl signals but not with pincer backbone protons (Figure S12).

## Conclusions

The unsaturated PNP complex **1^*L*2^** provides the second example of reductive, electrochemically driven N_2_ splitting. In analogy to parent **2^*L*1^**, Brønsted acid protonates the pincer backbone of N_2_‐derived nitride **2^*L*2^**, yet at a distinctly different site. However, this product may serve as starting platform for nitrogen incorporation into organic molecules as demonstrated by nitride methylation with MeOTf. A strong dependence of the nitrogen splitting yield on the nature of chemical reductants (CoCp*_2_: 60 %, Na/Hg: 30 %, KC_8_: 20 %) or electrolysis (15 %) was found, which markedly differs from parent **1^*L*1^** (CoCp*_2_: 75 %, Na/Hg: 80 %, electrolysis: 60 %). The unproductive decomposition pathways that diminish the yield in **2^*L*2^** were not examined in detail. However, detailed comparison of electrochemical data for **1^*L*2^** vs. parent **1^*L*1^** allowed for identifying three key differences that provide a qualitative basis to rationalize the trends in rhenium mediated N_2_ splitting yields with different pincer ligands and reductants:

a) Unlike **1^*L*1^**, the starting complex **1^*L*2^** exhibits slow decomposition in the presence of N_2_ and chloride ions. The decreased stability against chloride is partly attributed to decay via chloride‐induced disproportionation. The reduced chemical stability should affect electrosynthetic vs. chemical reduction yields which proceed on much slower timescales with concomitant free chloride buildup.

b) Weaker N→M π‐donation by pincer ligand *L*2 results in an anodic shift of the Re^III/II^ and Re^II/I^ redox couples and a smaller separation of their potentials. This allows for electrochemically driven N_2_ splitting at more desired, less negative potentials. However, unproductive Re^II/I^ reduction prior to N_2_ activation and additional *L*2 ligand fragmentation pathways via isobutene liberation might be more accessible with strong chemical reductants, such as Na/Hg or KC_8_, leading to decay due to over‐reduction.

c) In addition to the kinetic model proposed for **1^*L*2^**, a rapid bimolecular decay pathway was found for the key rhenium(II) species [ReCl_2_(*L*2)]^–^ that can compete with productive N_2_ activation. This pathway will be particularly detrimental for heterogeneous chemical (Na/Hg, KC_8_) and electrochemical reduction where high local Re^II^ concentrations are expected.

This study exemplifies the subtle interplay of the underlying thermodynamics and kinetics of electron transfer processes and coupled chemical steps, respectively, as determining parameters for the yields in reductive N_2_ splitting. Future work will have to focus on the nature of the decay pathways to design improved platforms for (electro‐)chemical N_2_ fixation.

## Experimental Section

Materials and Synthetic Methods

All experiments were carried out under inert conditions using standard Schlenk and glove‐box techniques under Ar or N_2_. HPLC grade solvents (Sigma Aldrich/Merck) were dried using an MBRAUN Solvent Purification System. THF was additionally dried with Na/K and chlorobenzene over CaH_2_. Deuterated solvents were bought from Euriso‐Top GmbH and dried with Na/K ([D_8_]THF) or 4 Å molecular sieves (C_6_D_6_). ^15^N_2_, Si(CH_3_)_3_N_3_, Co(Cp)_2_, Co(Cp*)_2_, hexamethylbenzene, P[OSi(CH_3_)_3_]_3_, PPh_3_O were used as purchased. HOTf and MeOTf were distilled prior to use. Na/Hg (1 m) was prepared from elemental Na and Hg. Fe(Cp)_2_ and Fe(Cp*)_2_ were sublimed and (*n*Bu_4_N)PF_6_, (*n*Bu_4_N)Cl, and (*n*He_4_N)Cl dried before use. KC_8_ was synthesized by layering metallic potassium (332 mg, 8.49 mmol, 1.15 equiv.) with graphite (mesh 335, 711 mg, 59.2 mmol, 8 equiv.) and heating under vacuum, until full intercalation and observation of the characteristic bronze color. 2,4,6‐tri‐*tert*‐butylphenoxy radical, **1^*L*1^**, and [ReCl_3_(*L*1)] were prepared according to published procedures.[12c], [12f], [12g], 27

Analytical Methods

Elemental analyses were obtained from the “Analytisches Labor” at University of Goettingen using an Elementar Vario EL 3 analyzer. NMR spectra were recorded on a Bruker Avance III 300, Avance III 400, or Avance 500 spectrometer with broadband cryoprobe and calibrated to the residual solvent signals (C_6_D_6_: *δ* ^1^H = 7.16 ppm, *δ* ^13^C = 128.4 ppm, [D_8_]THF: *δ* ^1^H = 3.58 ppm, *δ* ^13^C = 67.6 ppm, CD_2_Cl_2_: *δ* ^1^H = 5.32 ppm, *δ* ^13^C = 53.84 ppm). ^31^P NMR and ^15^N NMR chemical shifts are reported relative to external phosphoric acid and nitromethane standard (*δ* ^31^P = 0.0 ppm, *δ* ^15^N = 0.0 ppm), respectively. Signal multiplicities are abbreviated as: s (singlet), d (doublet), m (multiplet). UV/Vis absorption spectra were measured on a CARY300 Scan Varian spectrometer using inert sealed cuvettes. Liquid injection field desorption mass spectrommetry (LIFDI‐MS, JEOL AccuTOF JMS‐T100GCV) was measured at the “Zentrale Massenabteilung” at University of Goettingen. Electrochemical experiments were carried out with Metrohm PGSTAT101 (data under Ar) and GAMRY 600 reference (N_2_ data) potentiostats using standard software. CV was measured using glassy carbon (1.6 mm diameter) working and Pt wire counter electrodes and a Ag wire pseudo‐reference electrode in a fritted sample holder compartment and referenced against the [Fe(Cp)_2_]^+/0^ couple. CPE was performed using reticulated vitreous carbon as working electrode, Pt‐wire counter electrode in a fritted compartment with Fe(Cp*)_2_ as sacrificial reductant and a Ag‐wire as pseudo‐reference electrode in a fritted sample holder. For all electrochemical experiments, a 0.2 m (*n*Bu_4_N)PF_6_ solution in THF was used as electrolyte, with appropriate iR compensation. High‐pressure CV was carried out in a reactor as described previously.[12g] Magnetic susceptibility measurements were performed with a Quantum Design MPMS‐XL‐5 SQUID magnetometer in the temperature range from 295–2 K at 0.5 T applied field. Powdered samples were contained in Teflon buckets and fixed in a non‐magnetic sample holder. Each raw data point was corrected for diamagnetic contribution of the bucket by subtraction of its experimentally derived magnetic moment. The molar susceptibility data were corrected for the diamagnetic contribution using the Pascal constants and the increment method according to Haberditzl.^26^ Experimental data were modelled with the julX program.^27^ The diffraction data were obtained at 100 K on a Bruker D8 three‐circle diffractometer, equipped with a PHOTON 100 CMOS detector and an INCOATEC microfocus source with Quazar mirror optics (Mo‐*K*_α_ radiation, *λ*= 0.71073 Å).

https://www.ccdc.cam.ac.uk/services/structures?id=doi:10.1002/ejic.201901278 1832926 (for **2^*L*2^**) contains the supplementary crystallographic data for this paper. These data can be obtained free of charge from http://www.ccdc.cam.ac.uk/structures.

Synthetic and Electrochemical Experiments

**ReCl_2_(*L*2) (1^*L*2^): 1^*L*1^** (120 mg, 194 µmol, 1.0 equiv.) and the 2,4,6‐tri‐*tert*‐butylphenoxy radical (305 mg, 1.17 mmol, 6.0 equiv.) are mixed in benzene (15 mL) and stirred for 24 h at 50 °C. The solvent is removed in vacuo and the product is washed with excess pentane, until the washing solution is colourless. Co(Cp)_2_ (7 mg, 37 µmol, 0.2 equiv.) is added and the product is dissolved in benzene and stirred for 2 h at r.t. The reaction mixture is filtered, the benzene phase is lyophilized and remaining CoCp_2_ and 2,4,6‐tri‐*tert*‐butylphenol are sublimed off overnight at 75 °C. **1^*L*2^** is obtained as a brown powder in 63 % yield. Anal. Calcd for C_20_H_40_Cl_2_NP_2_Re (%): C, 39.15; H, 6.57; N, 2.28; found C, 38.81, H, 6.63; N, 2.14. NMR (C_6_D_6_, [ppm]): ^1^H (300 MHz): *δ* = 0.90 (d, ^3^ *J*_HH_ = 6.4 Hz, 2H, PCH), 2.61 (A_18_XX'A'_18_, *N* = |^3^ *J*_HP_+^5^ *J*_HP_| = 6.2 Hz, 36H, P(C(CH_3_)_3_)), 3.65 (A_2_B_2_XX'B_2_'A_2_', *N* = |^3^ *J*_HP_+^4^ *J*_HP_| = 16.4 Hz, ^3^ *J*_HH_ = 6.4 Hz, 2H, NCH). ^13^C (75.5 MHz): *δ* = 34.7 (A_6_XX'A'_6_, *N* = |^2^ *J*_CP_+^4^ *J*_CP_| = 2.5 Hz, P(C(CH_3_)_3_)), 77.4 (A_2_XX'A'_2_, *N* = |^1^ *J*_CP_+^3^ *J*_CP_| = 9.5 Hz, P(C(CH_3_)_3_)), 147.6 (AXX'A', *N* = |^1^ *J*_CP_+^3^ *J*_CP_| = 15.4 Hz, PCH), 212.4 (AXX'A', *N* = |^2^ *J*_CP_+^3^ *J*_CP_| = 7.9 Hz, NCH). ^31^P{^1^H} (121.5 MHz): *δ* = –275.6 (s). LIFDI‐MS (toluene, [*m/z*]): 613.1 (100 %, [M]^+^).

**Re(N)Cl(*L*2) (2^L2^):** *N_2_ route*. Degassed THF (0.45 mL) is vacuum‐transferred to a mixture of **1^*L*2^** (5.0 mg, 8.1 µmol, 1.0 equiv.) and reductant [Co(Cp^*^)_2_: 3.0 mg, 9.0 µmol, 1.1 eq; NaHg (1 m): 121.3 mg, 9.0 µmol, 1.1 eq or KC_8_: 1.1 mg, 8.1 µmol, 1.0 equiv.] in a J‐Young NMR tube and placed under an N_2_‐atmosphere. After thawing of the solvent, the mixture is shaken vigorously with gradual colour change from dark brown to light brown. After 30 min at r.t., the solvent is removed, hexamethylbenzene (1 eq via a 0.08 m stock solution in THF) is added and the solvent is removed again. Spectroscopic yields of the title compound are obtained by integration of the *L*2 ligand backbone ^1^H NMR signals vs. the internal standard C_6_Me_6_ [60 % for Co(Cp^*^)_2_; 30 % for Na/Hg; 20 % for KC_8_]. **2^*L*2^** was not isolated via this route.

*Azide route*. **1^*L*2^** (25.0 mg, 40.7 µmol, 1.0 equiv.) is dissolved in THF (1 mL) and added dropwise over a period of 5 min to a stirring solution of Me_3_SiN_3_ (26.78 µL, 23.5 mg, 203 µmol, 5.0 equiv.) in THF (0.5 mL). The solution is stirred at r.t. for 1.5 h after which the solvent is removed in vacuo. After extraction with pentane (4 × 5 mL) and removal of the solvent, **2^*L*2^** is obtained as a light brown solid in 72 % yield. Anal. Calcd. for C_20_H_40_ClN_2_P_2_Re (%): C, 40.57; H, 6.81; N, 4.73; found C, 40.66; H, 6.73; N, 5.01. NMR (C_6_D_6_, ppm): ^1^H (400 MHz): *δ* = 1.18 (A_9_XX'A'_9_, *N* = |^3^ *J*_HP_+^5^ *J*_HP_| = 7.2 Hz, 18H, P(C(CH_3_)_3_)), 1.49 (A_9_XX'A'_9_, *N* = |^3^ *J*_HP_+^5^ *J*_HP_| = 7.0 Hz, 18H, P(C(CH_3_)_3_)), 4.29 (A_2_B_2_XX'B_2_'A_2_', *N* = |^2^ *J*_HP_+^4^ *J*_HP_| = 2.2 Hz, ^3^ *J*_HH_ = 6.3 Hz, 2H, PCH), 7.00 (A_2_B_2_XX'B_2_'A_2_', *N* = |^3^ *J*_HP_+^5^ *J*_HP_| = 17.1 Hz, ^3^ *J*_HH_ = 6.2 Hz, 2H, NCH). ^13^C{^1^H} (125.76 MHz): *δ* = 28.5 (br, 6C, P(C(CH_3_)_3_)), 29.4 (br, 6C, P(C(CH_3_)_3_)), 34.9 (AXX'A', *N* = |^1^ *J*_CP_+^3^ *J*_CP_| = 10.3 Hz, 2C, P(C(CH_3_)_3_)), 36.7 (AXX'A', *N* = |^1^ *J*_CP_+^3^ *J*_CP_| = 11.8 Hz, 2C, P(C(CH_3_)_3_)), 91.8 (AXX'A', *N* = |^1^ *J*_CP_+^3^ *J*_CP_| = 20.9 Hz, 2C, PCH), 170.3 (AXX'A', *N* = |^2^ *J*_CP_+^4^ *J*_CP_| = 6.8 Hz, 2C, NCH). ^31^P{^1^H} (161.25 MHz): *δ* = 71.8 (s). LIFDI‐MS (toluene, [*m/z*]): 592.1 (100 %, [M]^+^).

**ReCl_3_(*L*2) (4^*L*2^):** ReCl_3_(*L*1) (15.3 mg, 0.024 mmol) and 2,4,6‐tri‐*tert*‐butylphenoxy radical (33.9 mg, 0.12 mmol, 5.4 equiv.) are combined in C_6_H_6_ (dried with Na/K). After heating at 60 °C for 1.5 h, the solvents are evaporated in vacuo. After extensive washing with pentane, and lyophilization (C_6_H_6_), **4^*L*2^** is obtained in 70 % yield (10.6 mg; 0.016µmol). Anal. Calcd. for C_20_H_40_Cl_3_NP_2_Re (%): C, 37.01; H, 6.21; N, 2.16; found C, 36.66; H, 6.29; N, 1.96. ^1^H NMR (300 MHz, C_6_D_6_): 15.2 ppm (s, Δ_v1/2_ = 7.5 Hz), –51.7 (s, Δ_v1/2_ = 14.8 Hz), –194.6 (s, Δ_v1/2_ = 30.3 Hz). LIFDI‐MS (Toluene, [*m/z*]): 648.1 (100 %, [M]^+^), calculated 648.1.

**[ReNCl(H*L*2)]OTf (5^H*L*2^‐OTf): 2^*L*2^** (5.0 mg, 8.4 µmol, 1.0 equiv.) is dissolved in Et_2_O (1 mL) and HOTf (0.74 µL, 8.4 µmol, 1 equiv.) is added via an Eppendorf pipette. Upon stirring for 1 h, a red‐brownish precipitate forms which is collected by filtration, washed with pentanes (3 × 2 mL) and dried in vacuo to give **5^HL2^‐OTf** in 82 % yield. NMR (CD_2_Cl_2_, ppm) ^1^H (500 MHz): *δ* = 1.28 (d, ^3^ *J*_HP_ = 14.8 Hz, 9H, CH_3_), 1.32 (d, ^3^ *J*_HP_ = 14.7 Hz, 9H, CH_3_), 1.57 (d, ^3^ *J*_HP_ = 15.9 Hz, 9H, CH_3_), 1.60 (d, ^3^ *J*_HP_ = 15.6 Hz, 9H, CH_3_), 3.79 (dd, ^2^ *J*_HH_ = 21.2 Hz, ^2^ *J*_HP_ = 7.7 Hz, 1H, P‐CH_2_‐CH), 4.40 (dd, ^2^ *J*_HH_ = 21.2 Hz, ^2^ *J*_HP_ = 7.4 Hz, 1H, P‐CH_2_‐CH), 6.66 (d, ^3^ *J*_HH_ = 6.7 Hz, 1H, P‐CH), 8.02 (dd, ^3^ *J*_HP_ = 27.8 Hz, ^3^ *J*_HH_ = 6.6 Hz, 1H, N‐CH=CH), 9.35 (d, ^3^ *J*_HP_ = 20.9 Hz, 1H, N=CH‐CH_2_). ^13^C{^1^H} (125.7 MHz): *δ* = 28.6 (d, ^2^ *J*_CP_ = 3.5 Hz, CH_3_), 28.7 (d, ^2^ *J*_CP_ = 4.1 Hz, CH_3_), 29.0 (d, ^2^ *J*_CP_ = 3.9 Hz, CH_3_), 29.1 (d, ^2^ *J*_CP_ = 3.3 Hz, CH_3_), 36.0 (d, ^1^ *J*_CP_ = 18.1 Hz, C(CH_3_)_3_), 36.3 (d, ^1^ *J*_CP_ = 15.1 Hz, C(CH_3_)_3_), 37.8 (dd, ^1^ *J*_CP_ = 15.5 Hz, ^3^ *J*_CP_ = 3.9 Hz, C(CH_3_)_3_), 38.2 (dd, ^1^ *J*_CP_ = 18.7 Hz, ^3^ *J*_CP_ = 3.7 Hz, C(CH_3_)_3_), 41.0 (d, ^1^ *J*_CP_ = 22.5 Hz, CH_2_‐P), 127.9 (d, ^1^ *J*_CP_ = 31.9 Hz, CH‐P), 163.9 (d, ^2^ *J*_CP_ = 3.8 Hz, N‐CH=CH), 201.2 (s, N=CH‐CH_2_). ^31^P{^1^H} (121.5 MHz): *δ* = 70.0 (d, ^2^ *J*_PP_ = 148.1 Hz), 73.0 (d, ^2^ *J*_PP_ = 148.1 Hz).

**[Re(NMe)Cl(*L*2)]OTf (6^*L*2^‐OTf): 2^*L*2^** (25.0 mg, 44.2 µmol, 1.0 equiv.) and MeOTf (5.26 µL, 46.4 µmol, 1.1 equiv.) are dissolved in chlorobenzene and heated to 80 °C for 12 h. After removal of all volatiles in vacuo, the product is washed with Et_2_O and **6^*L*2^‐OTf** is obtained as brown solid in 80.5 % yield. Anal. Calcd. (%): C, 34.94; H, 5.71; N, 3.70; found C, 35.18; H, 5.71; N, 3.49. NMR (CD_2_Cl_2_, ppm): ^1^H (500 MHz): *δ* = 1.29 (A_9_XX'A'_9_, *N* = |^3^ *J*_HP_ + ^5^ *J*_HP_| = 6.6 Hz, 18H, PC(CH_3_)_3_), 1.49 (A_9_XX'A'_9_, *N* = |^3^ *J*_HP_ + ^5^ *J*_HP_| = 7.8 Hz, 18H, PC(CH_3_)_3_), 2.70 (s_br_, 3H, NCH_3_), 5.34 (m, 2H, PCH), 7.99 (A_2_B_2_XX'B'_2_A'_2_, *N* = |^3^ *J*_HP_ + ^4^ *J*_HP_| = 18.2 Hz, ^3^ *J*_HH_ = 6.5 Hz, 2H, NCH). ^13^C{^1^H} (125.7 MHz): *δ* = 28.9 (s, PC(CH_3_)_3_), 30.2 (A_3_XX'A'_3_, *N* = |^2^ *J*_CP_ + ^4^ *J*_CP_| = 2.0 Hz, PC(CH_3_)_3_), 39.6 (AXX'A', *N* = |^1^ *J*_CP_ + ^3^ *J*_CP_| = 11.6 Hz, PC(CH_3_)_3_), 40.2 (AXX'A', *N* = |^1^ *J*_CP_ + ^3^ *J*_CP_| = 9.7 Hz, PC(CH_3_)_3_), 61.9 (s, NCH_3_), 99.1 (AXX'A', ^1^ *J*_CP_ = 22.6 Hz, ^3^ *J*_CP_ = 20.4 Hz, PCH), 172.9 (AXX'A', *N* = |^2^ *J*_CP_ + ^3^ *J*_CP_| = 5.3 Hz, NCH). ^31^P{^1^H}: (202.4 MHz) *δ* = 88.8 (s). LIFDI (toluene, *m/z*) = 607.2 (100 %, [M^+^]).

**Chemical stability tests of 1^*L*2^**: **1^*L*2^** (3.0 mg; 5.0 µmol) was dissolved in THF (0.6 mL) in a J‐Young tube under Argon and the stability was monitored by NMR spectroscopy. The sample was degassed by three freeze‐pump‐thaw cycles and backfilled with N_2_ and the stability was again monitored by NMR spectroscopy over time. To examine the stability in the presence of chloride, a sample with added (*n*Bu_4_N)Cl (6.5 mg; 23.5 µmol; 5 equiv.) was monitored by NMR spectroscopy. NMR spectra are depicted as Figure S15 and Figure S17.

**Controlled potential electrolysis: 1^*L*2^** (2.6 mg, 4.2 µmol) and 4 mL of 0.2 m (*n*Bu_4_N)PF_6_ electrolyte solution in THF was added to the working electrode compartment of the electrolysis cell. The solution was electrolyzed for 2 h at the peak potential of the first reduction feature obtained by CV, resulting in a colour change from light brown to green. Integration of the current vs. time plot gave a charge corresponding to 1.2 mol e^–^ per mol Re. The solvent was evaporated to give a light green solid, which was dissolved in 0.6 mL of THF. PPh_3_O (3.2 mg, 11.5 µmol) was added as internal standard, and the yield in Re(N)Cl(*L*2) (**2^L2^**) (17 %) was derived by ^31^P{^1^H} NMR spectroscopically in C_6_D_6_, see Figure S8.

**Chloride concentration dependent CV under Ar: 1^*L*2^** (2.5 mg, 4.0 µmol) was dissolved in a 0.2 m solution of (*n*Bu_4_N)PF_6_ in THF (4 mL) and a small amount of Fe(Cp*)_2_ was added as an electrochemical reference. In sequence, equivalents of (*n*Bu_4_N)Cl (1.1 mg, 1 equiv.; 1.1 mg, 2 eq total; 3.3 mg, 5 eq total; 5.5 mg, 10 equiv. total; 11.1 mg, 20 equiv. total) were added. After each chloride addition, CV's were recorded quickly at 0.5, 1, 2, 3, 4, and 5 V s^–1^ only under Ar, before **1^*L*2^** shows substantial decomposition (see Figure 2).

**Rhenium concentration dependent CV under Ar:** A stock solution of **1^*L*2^** was prepared by dissolving **1^*L*2^** (15.3 mg, 25 µmol) in a 1.0 mL of solution of 0.2 m (*n*Bu_4_N)PF_6_ in THF. Aliquots of this stock solution were added to a 5 mL of solution of 0.2 m (*n*Bu_4_N)PF_6_ in THF, with a spatula tip of Fe(Cp)_2_ as an electrochemical reference, to afford solutions of 0.5, 1.0, 2.0, 3.0 and 4.0 mm **1^*L*2^**. CVs for both the first two reduction features were recorded at 0.1 V s^–1^ (see Figure S20).

**N_2_‐pressure dependent CV: 1^*L*2^** (2.5 mg, 4.0 µmol) was dissolved in a 0.2 m solution of (*n*Bu_4_N)PF_6_ in THF (4 mL) and a small amount of Fe(Cp*)_2_ was added as an electrochemical reference. The solution was transferred to the Parr reactor and subsequently pressurized with N_2_ to obtain CVs at 1, 3, 5, 7, 9, 11 bars. At 11 bars, the system was allowed to stay for 45 minutes while regular CVs were measured (see Figures S20). After depressurzing, the reactor was transferred back in the glovebox and the reaction mixture was analysed by ^31^P{^1^H} NMR spectroscopy (see Figure S16).

## Supporting information

Supporting Information
