# Supplementary material for: (Electro‐)chemical Splitting of Dinitrogen with a Rhenium Pincer Complex
Source: Eur J Inorg Chem. 2020 Jan 31;2020(15-16):1402–10. doi: 10.1002/ejic.201901278 (PMC7217231; doi:10.1002/ejic.201901278)
Supplement: Supplementary file 1 — Supporting Information [file EJIC-2020-1402-s001.pdf]

European Journal of Inorganic Chemistry

Supporting Information

(Electro-)chemical Splitting of Dinitrogen with a Rhenium Pincer Complex

Richt S. van Alten, Florian Wätjen, Serhiy Demeshko, Alexander J. M. Miller, Christian Würtele, Inke Siewert,* and Sven Schneider*

1. Spectroscopic results……………………………………………………………..……..S2

*1.1 $ReCl_2$(L2) (**1^{L2}**)*……………………………………………………………………...S2

*1.2. Re(N)Cl(L2) (**2^{L2}**)*…………………………………………………………………S3

*1.3. $ReCl_3$(L2) (**4**)* …………………………………………………………………...S5

*1.4. [ReNCl(HL2)]OTf (**5^{HL2}-OTf**)* ……………………………………………………S5

*1.5. [Re(NMe)Cl(L2)]^{OTf} (**6^{L2}-OTf**)*…………………………………………………S6

*1.6 NMR spectra of isobutene formation upon reduction of **1^{L2}** with Na/Hg*………………...S7

*1.7. NMR Spectra of **1^{L2}** under N_2 atmosphere and in presence of chloride*………………S7

2. CV Data………………………………………………………………………………S9

*2.1 $ReCl_2$(L2) (**1^{L2}**)*……………………………………………………………………..S9

*2.2. Re(N)Cl(L2) (**2^{L2}**)*…………………………………………………………………S10

2.3 $ReCl_3$(L2)] (4^{L2})… ……………………………………………………………S11

*2.4 [ReNCl(HL2)]^{OTf} (**$5^{HL2\ OTf}$**)*………………………………………………………S11

3. CV Simulations……………………………………………………………………..S12

4. SQUID magnetometry……………………………………………………………..S13

5. Decompostion Equilibrium Constant Calculation………………………………S14

6. Crystallographic Details…………………………………………………………..S16

6.1. General crystallographic experimental details……………………………………S16

*6.2. Crystallographic details of **2^{L2}***…………………………………………………….S17

7 References……………………………………………………………………………S23

1Spectroscopic Results

1.1 $ReCl_2(L2)$ ($\mathbf{1}^{L2}$)

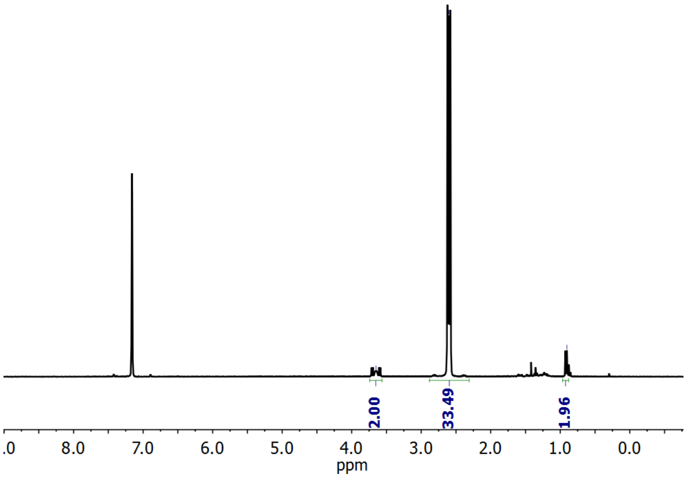

Figure S1. ^{1}H NMR spectrum of $\mathbf{1}^{L2}$ in C_6D_6.

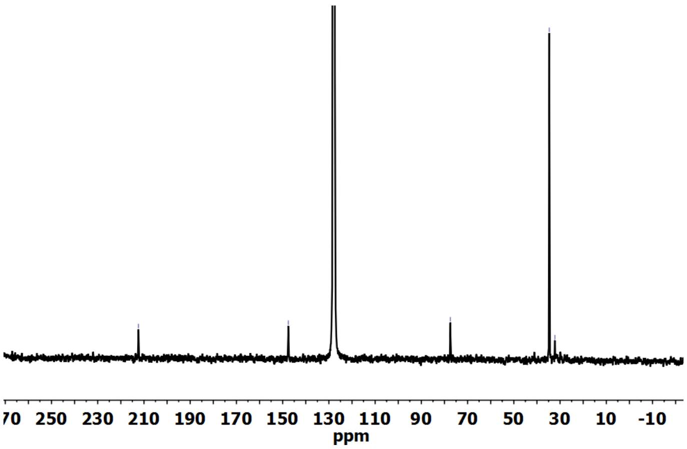

Figure S2. $^{13}C\{^{1}H\}$ NMR spectrum of $\mathbf{1}^{L2}$ in C_6D_6.

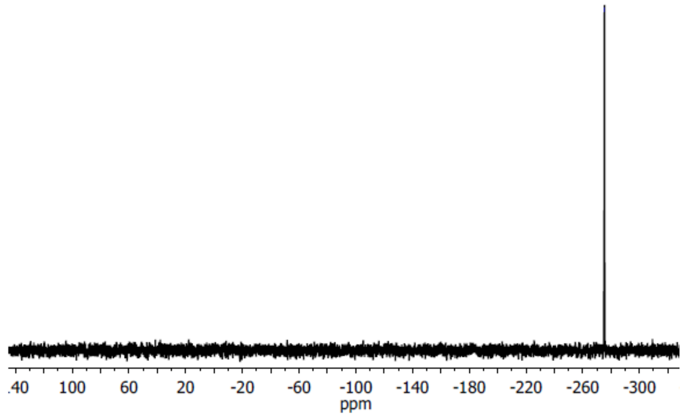

Figure S3. $^{31}P\{^{1}H\}$ NMR spectrum of $\mathbf{1}^{L2}$ in C_6D_6.

*1.2. Re(N)Cl(L2) (**2**^{L2})*

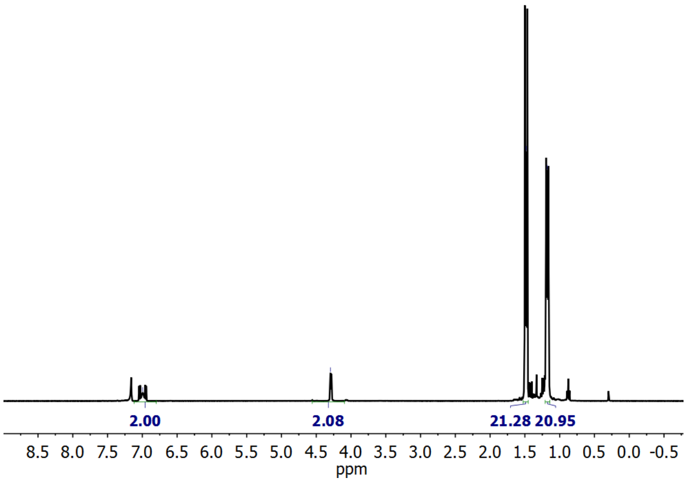

Figure S4. ^{1}H NMR spectrum of **2**^{L2} in C_6D_6.

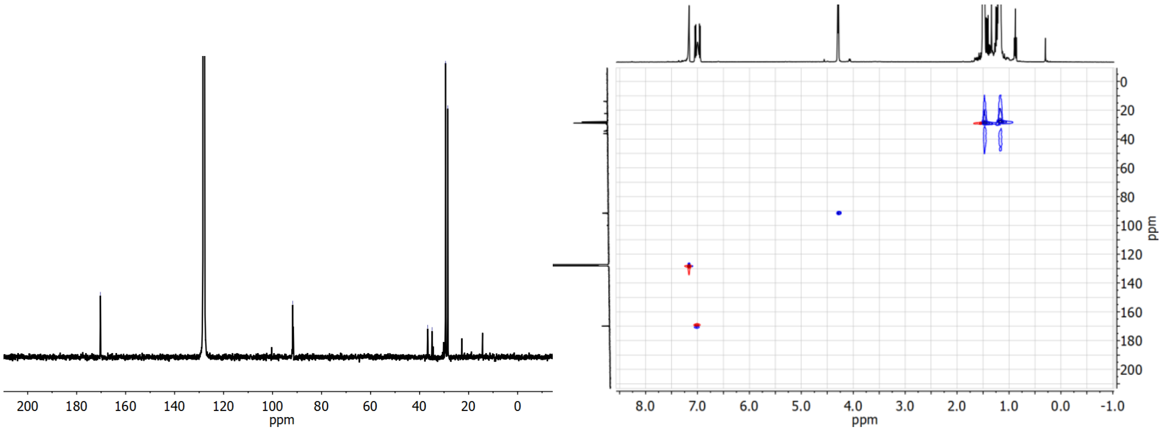

Figure S5. ^{13}C{^{1}H} NMR (*left*) and ^{1}H-^{13}C HSQC (*right*) spectra of **2**^{L2} in C_6D_6.

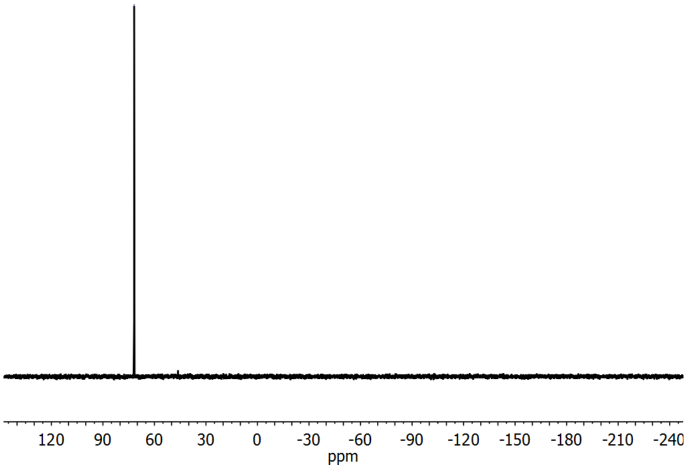

Figure S6. ^{31}P{^{1}H} NMR spectrum of **2**^{L2} in C_6D_6.

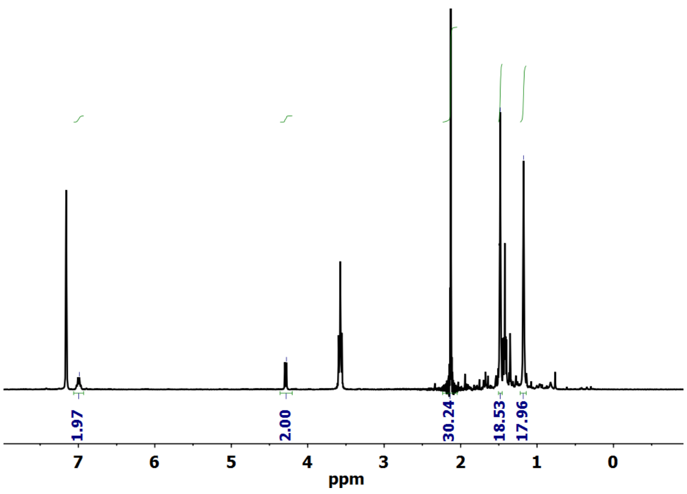

Figure S7. ^{1}H NMR spectrum of $\mathbf{2}^{L2}$ from reduction of $\mathbf{1}^{L2}$ with $Co(Cp^*)_2$ under N_2 using hexamethylbenzene as internal standard (@ 2.1 ppm).

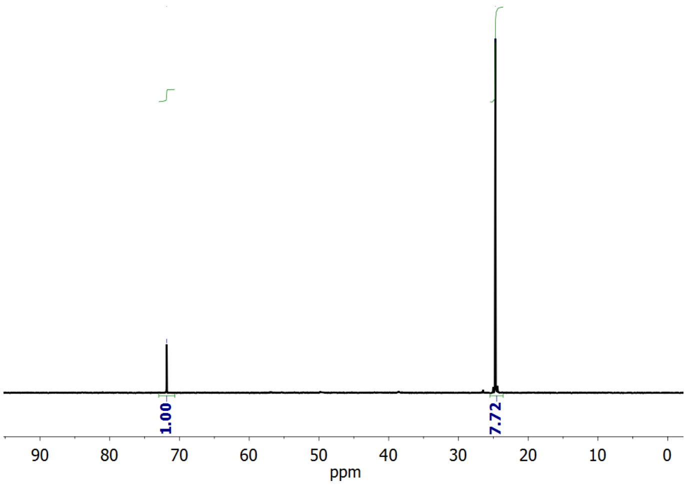

Figure S8. $^{31}P\{^1H\}$ NMR spectrum of $\mathbf{2}^{L2}$ from electrolysis of $\mathbf{1}^{L2}$ under N_2 using PPh_3O as internal standard (@ 24.7 ppm).

*1.3. $ReCl_3$(L2) (**4^{L2}**)*

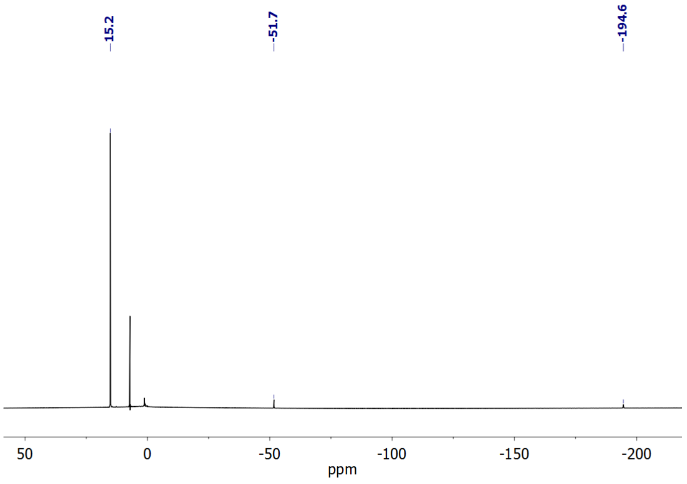

Figure S9. ^{1}H NMR spectrum of **4^{L2}** in C_6D_6.

*1.4. [ReNCl(HL2)]OTf (**5^{HL2}-OTf**)*

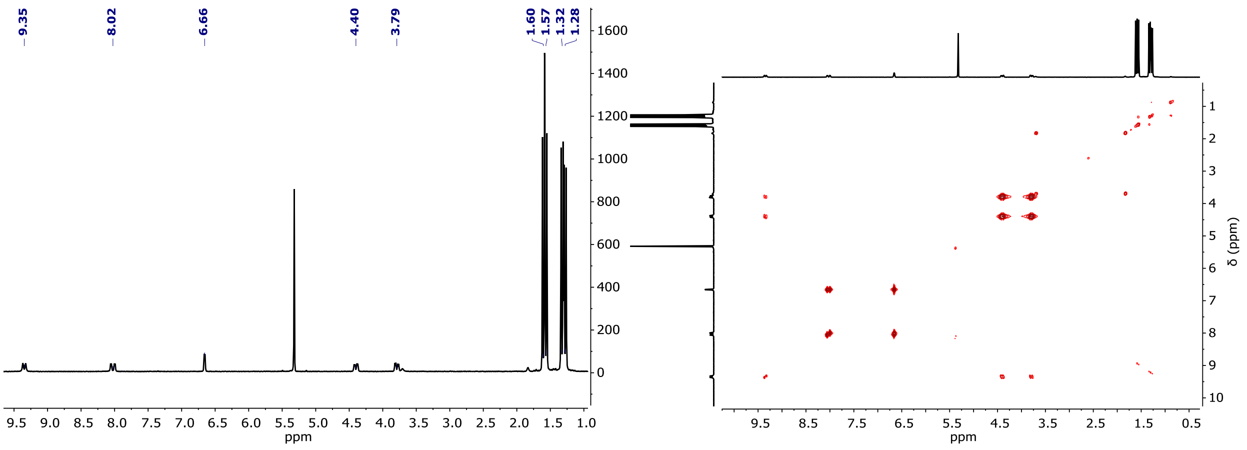

Figure S10. ^{1}H NMR (*left*) ^{1}H-^{1}H-COSY NMR (*right*) spectra of **5^{HL2}-OTf** in CD_2Cl_2.

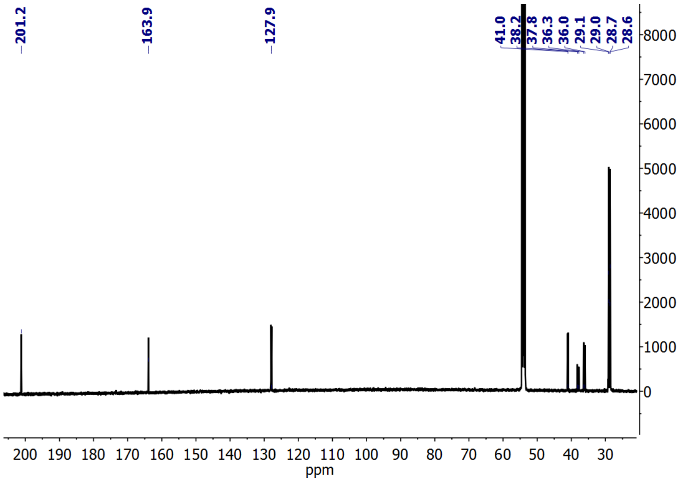

Figure S11 $^{13}C\{^{1}H\}$ NMR spectrum of $\mathbf{5^{\mathit{HL2}}}$**-OTf** in CD_2Cl_2.

*1.5. [Re(NMe)Cl(L2)]^{OTf} ($\mathbf{6^{\mathit{L2}}}$**-OTf**)*

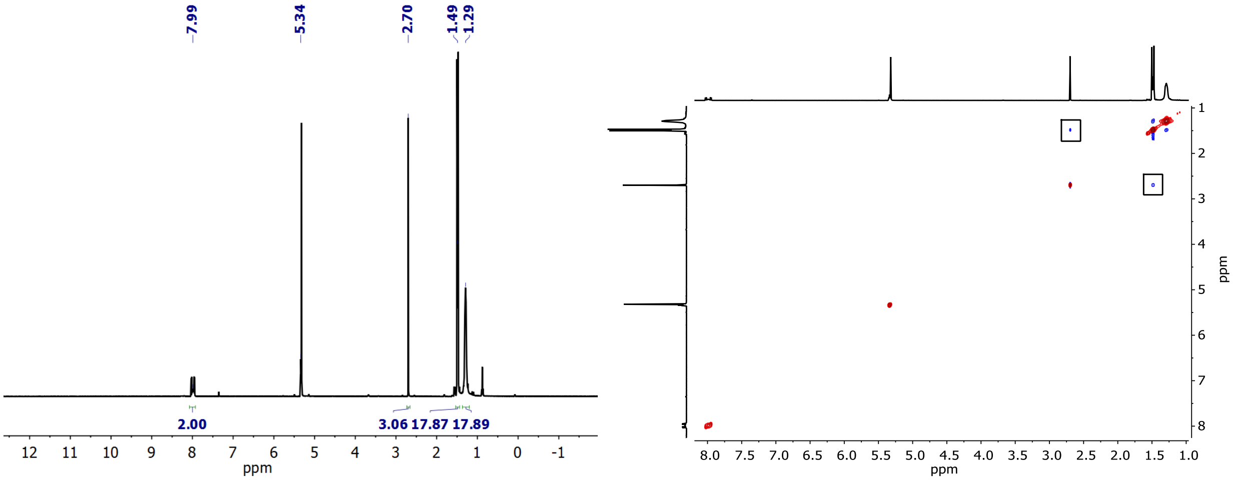

Figure S12. ^{1}H NMR (*left*) and ^{1}H-^{1}H-NOESY NMR (*right*) spectra of $\mathbf{6^{\mathit{L2}}}$**-OTf** in CD_2Cl_2.

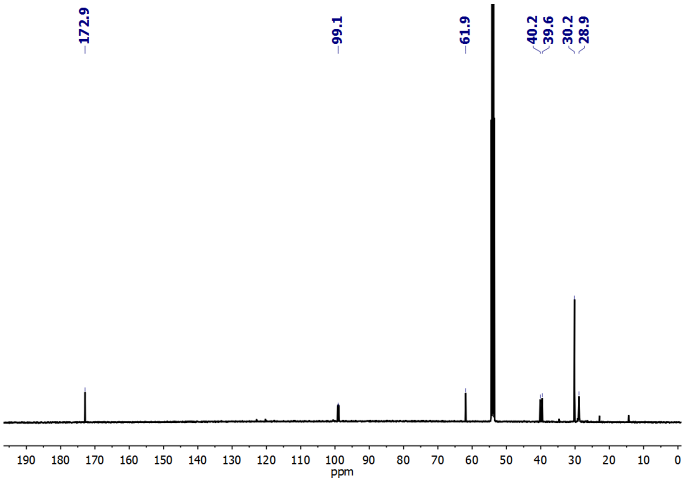

Figure S13. $^{13}C\{^1H\}$ NMR of $\mathbf{6}^{L2\ OTf}$ in CD_2Cl_2.

1.6 NMR spectra of isobutene formation upon reduction of $\mathbf{1}^{L2}$ *with Na/Hg*

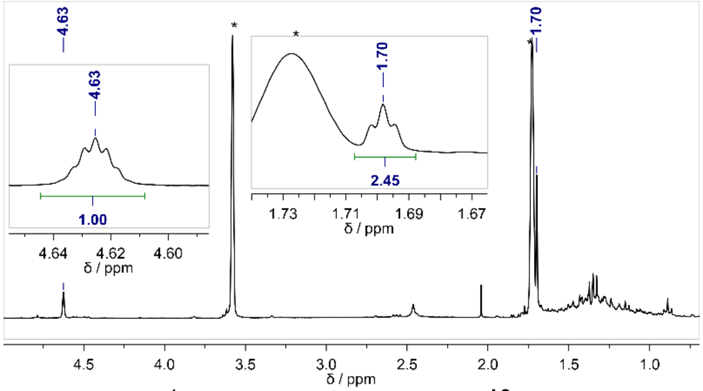

Figure S14. ^1H NMR of reduction of $\mathbf{1}^{L2}$ with 1 eq Na/Hg without N_2 showing isobutene formation. The isobutene liberation is also observed under N_2 atmosphere.

1.7. NMR Spectra of $\mathbf{1}^{L2}$ *under* N_2 *atmosphere and in presence of chloride.*

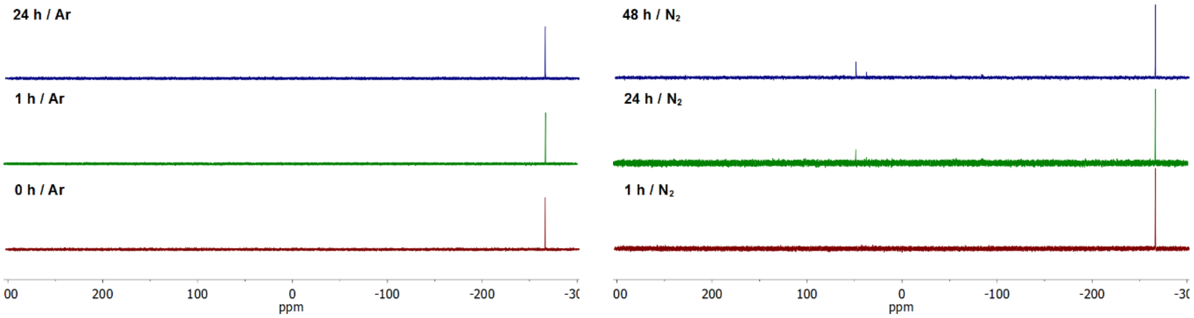

Figure S15. Stability of $\mathbf{1}^{L2}$ in THF-d_8 monitored by $^{31}P\{^1H\}$ NMR spectroscopy. *Left*: under Ar after 0 h, 1 h and 24 h *Right*: Under N_2 after 1 h, 24 h and 48 h.

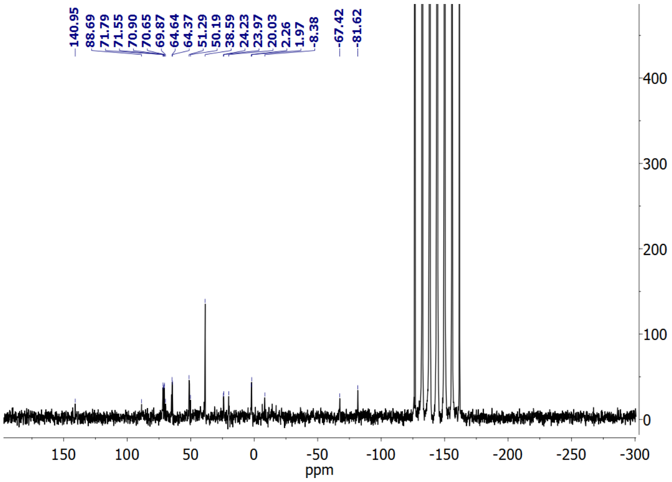

Figure S16. $^{31}P\{^{1}H\}$ NMR of the reaction mixture after measuring CV of $\mathbf{1}^{\boldsymbol{L2}}$ at increasing N_2-pressures (1, 3, 5, 7, 9, and 11 bars) and remaining at 11 bars of N_2 for 45 minutes and subsequent depressurizing in THF with 0.2 M ($^{n}Bu_4N$)PF_6.

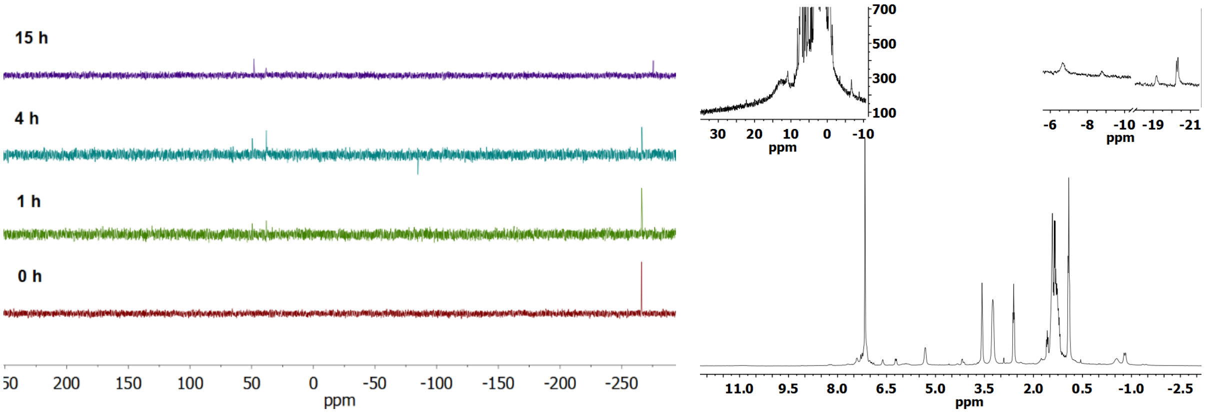

Figure S17. *Left*: $^{31}P\{^{1}H\}$ NMR spectra of $\mathbf{1}^{\boldsymbol{L2}}$ under N_2 in presence of ($^{n}Bu_4N$)Cl; the bottom three spectra measured were measured in THF and the top spectrum in C_6D_6. *Right*: ^{1}H NMR spectrum that refers to the top ^{31}P NMR spectrum the left side.

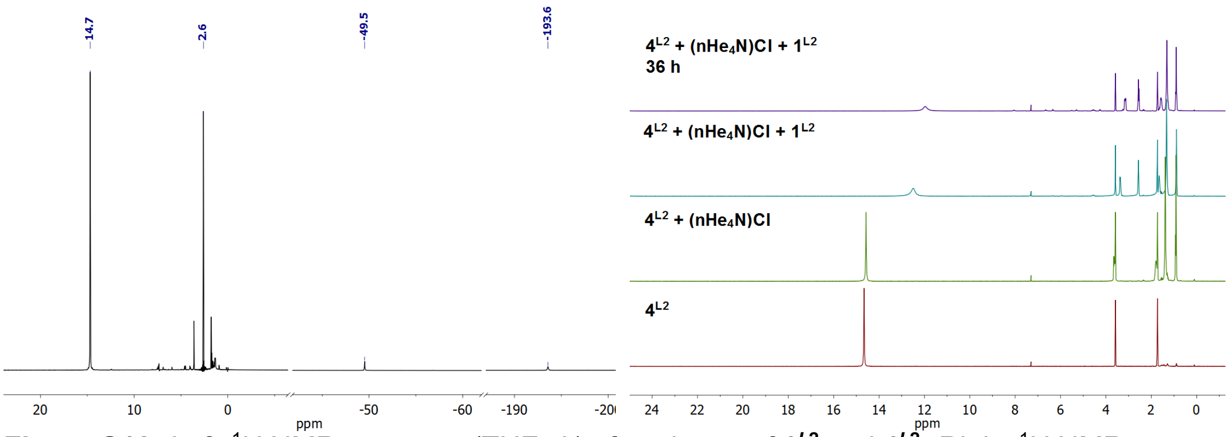

Figure S18. *Left*: ^{1}H NMR spectrum (THF-d_8) of a mixture of **1*^{L2}*** and **4*^{L2}***. *Right*: ^{1}H NMR spectrum (THF-d_8) of (from bottom to top): **4*^{L2}***, addition of 1 eq. of (nHe$_4$N)Cl, addition of 1 eq. of **1*^{L2}***, and the same reaction mixture after 36 h. Both experiments were performed under Ar.

2. CV Data

*2.1 ReCl$_2$(L2) (**1^{L2}**)*

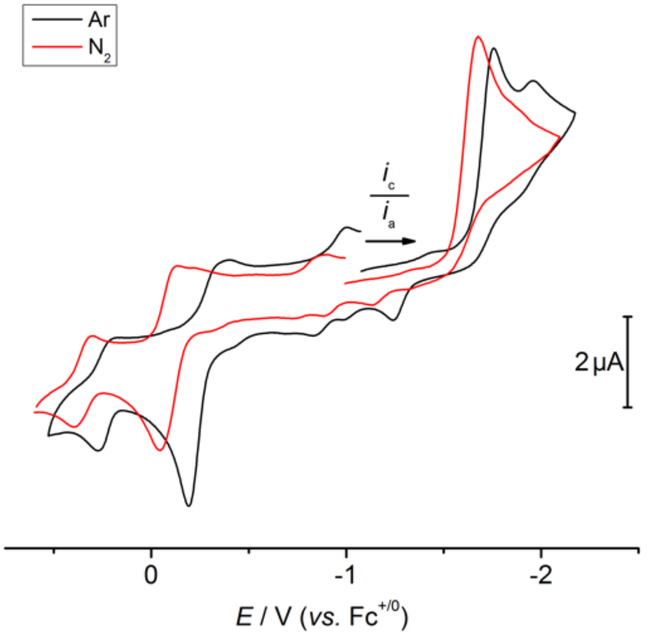

Figure S19. CV (v = 0.1 Vs^{-1}) of **1*^{L2}*** (1.0 mM in THF with 0.2 M (^{n}Bu$_4$N)PF$_6$) under N_2 (red) and Ar (black), respectively.

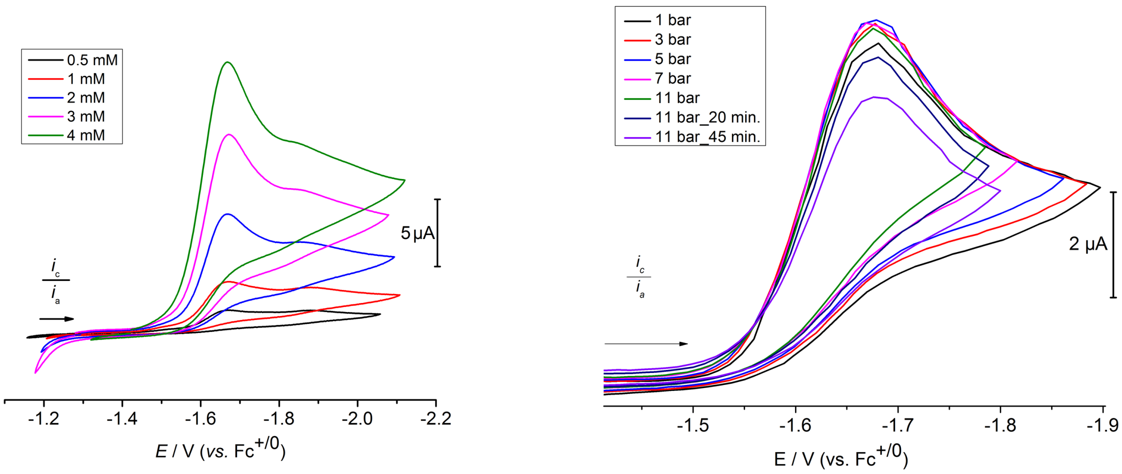

Figure S20. *Left*: CV (v = 0.1 Vs^{-1}) of $\mathbf{1}^{L2}$ (0.5-4.0 mM in THF with 0.2 M ($^{n}Bu_4N)PF_6$) under Ar. *Right*: CV (v = 0.1 Vs^{-1}) of $\mathbf{1}^{L2}$ (1.0 mM in THF with 0.2 M ($^{n}Bu_4N)PF_6$) at increasing pressures of N_2.

*2.2. Re(N)Cl(L2) (**2**^{L2})*

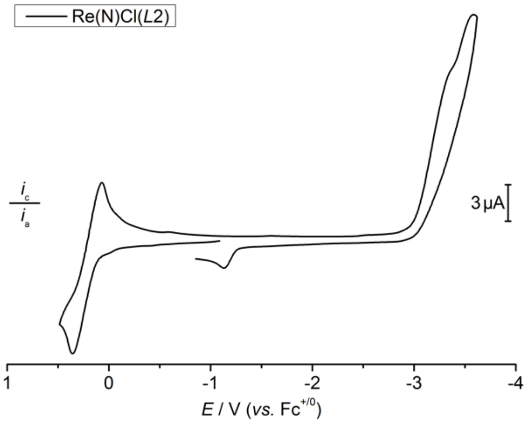

Figure S21. CV (v = 0.1 Vs^{-1}) of $\mathbf{2}^{L2}$ (1 mM in THF with 0.2 M ($^{n}Bu_4N)PF_6$).

2.3. ReCl$_3$(L2) (***4***^{L2})

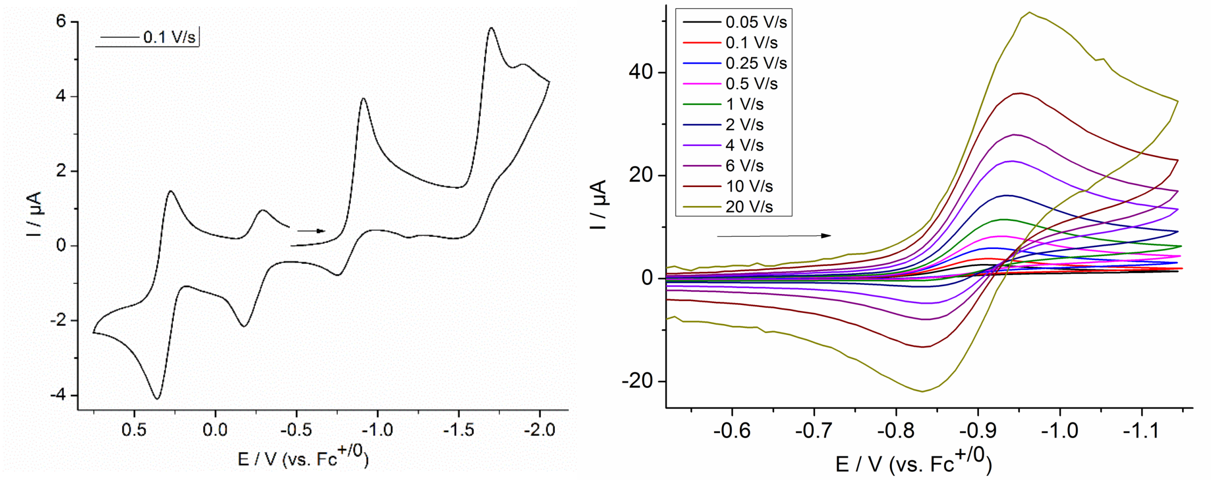

Figure S22. CV (v = 0.1 Vs^{-1}) of **4^{L2}** (1 mM in THF with 0.2 M (^{n}Bu$_4$N)PF$_6$) under Argon, *Right*: First reduction wave at different scan rates.

2.4 [ReNCl(HL2)]^{OTf} (***5***$^{HL2\ OTf}$)

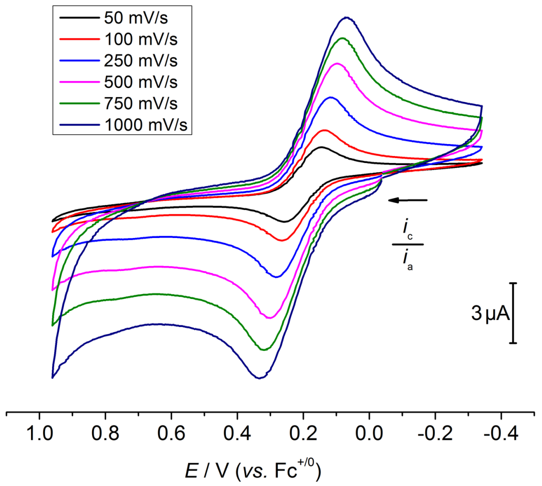

Figure S23. CV of **5^{HL2}-OTf** (1 mM in THF with 0.2 M (^{n}Bu$_4$N)PF$_6$).

3. CV Simulations

The diffusion coefficient of $\mathbf{1}^{L2}$ was determined as $D = 9.6 \cdot 10^{-6}$ cm²s^{-1} by DOSY NMR spectroscopy. The diffusion coefficient of all Re species was set to this value assuming small variation of D upon reduction or chloride. The diffusion coefficient of chloride ions was set to D = $5 \cdot 10^{-5}$ cm²s^{-1}.

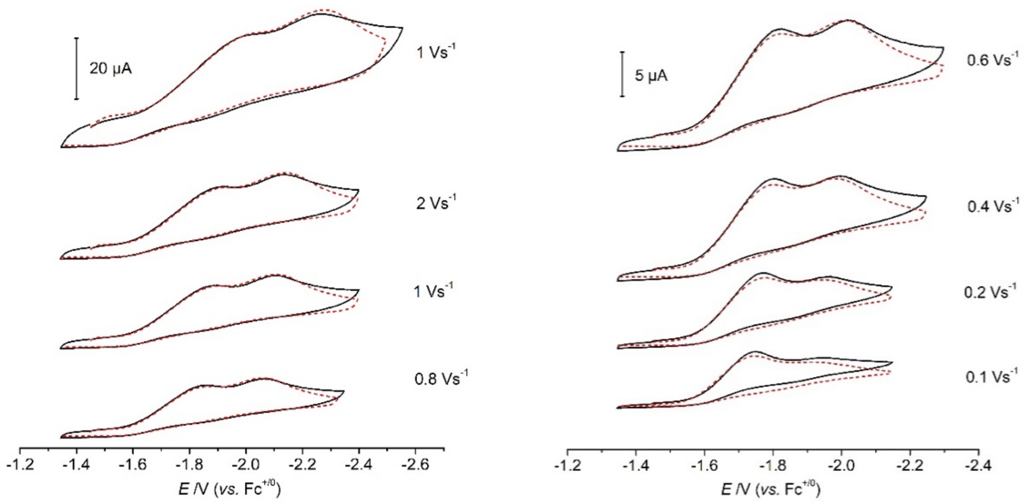

Figure S24. Experimental (black lines) and simulated (red dashed lines) scan-rate dependent CV data of $\mathbf{1}^{L2}$ under Ar in THF with 0.2 M (^{n}Bu$_4$N)PF$_6$; mechanism and simulation parameters according to Scheme 5.

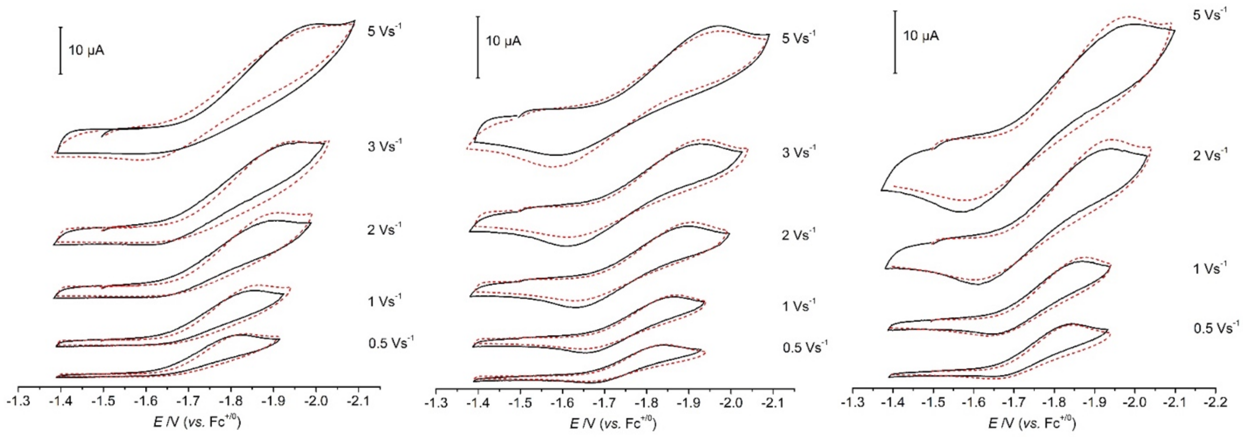

Figure S25. Experimental (black lines) and simulated (red dashed lines) CV data of $\mathbf{1}^{L2}$ under Ar in THF with 0.2 M (^{n}Bu$_4$N)PF$_6$ and different chloride ion concentrations; mechanism and simulation parameters according to Scheme 5. *Left*: 0 eq. [^{n}Bu$_4$N]Cl. *Middle*: 10 eq. of [^{n}Bu$_4$N]Cl. *Right*: 20 eq. [^{n}Bu$_4$N]Cl.

4. SQUID magnetometry

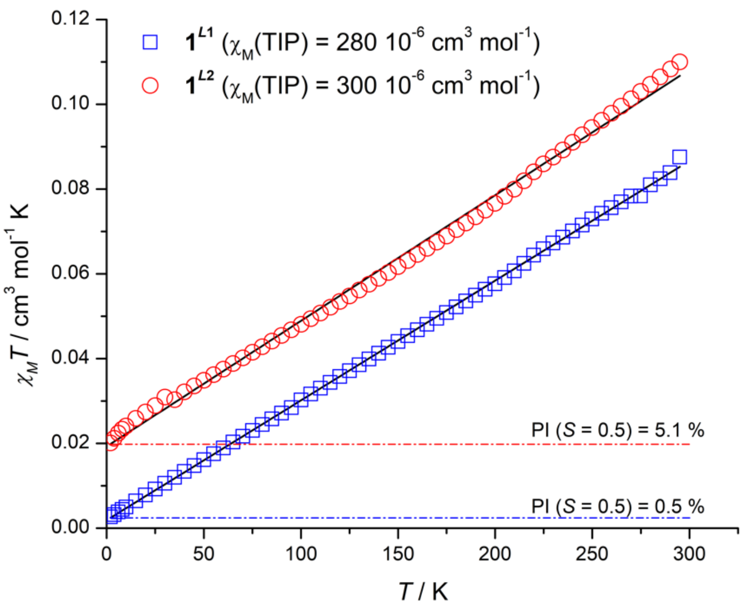

Figure S26. $\chi_M T$ *vs T* plot for $\mathbf{1}^{L1}$ and $\mathbf{1}^{L2}$ derived by SQUID magnetometry. The open circles and squares denote experimental susceptibility data and the black solid lines the best fits including paramagnetic impurities (PI with S = 0.5) of 0.5 % and 5.1 %, respectively.

5. Decomposition Equilibrium Constant Calculation

The thermochemistry for the chloride induced disproportionation of $\mathbf{1}^{\boldsymbol{L2}}$:

$$\underset{\mathbf{1}^{L2}}{[\mathrm{Re^{III}Cl_2}(L2)]} + \mathrm{Cl^-} \underset{K_{\mathrm{Cl}}}{\rightleftharpoons} [\mathrm{Re^{III}Cl_3}(L2)]^- \underset{K_{\mathrm{Disp.}}}{\overset{\mathbf{1}^{L2}}{\rightleftharpoons}} \underset{\mathbf{4}^{L2}}{[\mathrm{Re^{IV}Cl_3}(L2)]} + [\mathrm{Re^{II}Cl_2}(L2)]^-$$

was estimated from calculating the chloride association equilibrium constant K_{Cl} and the disproportionation constant $K_{\mathrm{Disp.}}$ as follows:

Calculation of K_{Cl}

The ^{31}P chemical shift of $\mathbf{1}^{\boldsymbol{L2}}$ is unchanged in the presence of approx. 5 eq. of $(\mathrm{nBu_4N})\mathrm{Cl}$. Furthermore, the absence of a new ^{31}P signal assignable to $[\mathrm{ReCl_3}(L2)]^-$ allows for estimating an upper limit for K_{Cl} assuming slow chloride exchange. An upper detection limit for $[\mathrm{ReCl_3}(L2)]^-$ is determined to be approximately 3 mM, based on a signal-to-noise ratio of 3:1 (see Figure S17).

$$K_{\mathrm{Cl}} = \frac{\mathrm{c}([\mathrm{ReCl_3}(L2)]^-)}{c([\mathrm{ReCl_2}(L2)]^-) * \mathrm{c}(\mathrm{Cl^-})}$$

$\mathrm{c}([\mathrm{ReCl_2}(L2)])_{\mathrm{starting}} = 8.3$ mM

$\mathrm{c}(\mathrm{Cl^-})_{\mathrm{starting}} = 39.1$ mM

$\mathrm{c}([\mathrm{ReCl_3}(L2)]^-)_{\text{upper limit}} \sim 3$ mM

$\mathrm{c}([\mathrm{ReCl_2}(L2)])_{\mathrm{limit}} \sim 5.3$ mM

$\mathrm{c}(\mathrm{Cl^-})_{\mathrm{limit}} \sim 36.1$ mM

$$K_{\mathrm{Cl}} < 0.016\ M^{-1}$$
$$\Delta G^0{}_{\mathrm{Cl}} \geq 2.5\ \mathrm{kcal\ mol^{-1}}$$

Calculation of K_{Disp}:

An upper limit for the disproportionation constant K_{Disp} of $\mathbf{1}^{L2}$ and $[Re^{III}Cl_3(L2)]^-$ to $\mathbf{4}^{L2}$ and $[Re^{II}Cl_2(L2)]^-$ was evaluated using the Re^{III}/Re^{II} reduction potential of $\mathbf{1}^{L2}$ as derived from digital simulation (see Scheme 5) and the Re^{IV}/Re^{III} reduction potential of $\mathbf{4}^{L2}$ derived by CV as an almost reversible wave at a scan rate of 20 V·s^{-1} (Figure S22):

$$K_{\mathrm{Disp}} = e^{\frac{n_1 n_2 F(E^0_{\mathbf{1}^{L2}} - E^0_{\mathbf{4}^{L2}})}{RT}}$$

$E^0_{\mathbf{1}^{L2}}$ = –1.75 V *vs* $Fc^{+/0}$

$E^0_{\mathbf{4}^{L2}}$ ≈ –0.9 V *vs* $Fc^{+/0}$

$$K_{\mathrm{Disp}} \approx 4 \cdot 10^{-15}$$

$$\Delta G^0_{Disp} \approx 20\ kcal\ mol^{-1}$$

5. Crystallographic Details

5.1. General crystallographic experimental details

CCDC-1832926 (**2**^{L2}) contains the supplementary crystallographic data for this paper. This data can be obtained free of charge via http://www.ccdc.cam.ac.uk/ products/csd/request/ (or from Cambridge Crystallographic Data Centre, 12 Union Road, Cambridge, CB2 1EZ, UK. Fax: +44-1223- 336-033; e-mail: deposit@ccdc.cam.ac.uk). Suitable single crystals for X-ray structure determination were selected from the mother liquor under an inert gas atmosphere and transferred in protective perfluoro polyether oil on a microscope slide. The selected and mounted crystals were transferred to the cold gas stream on the diffractometer. The diffraction data were obtained at 100 K on a Bruker D8 three-circle diffractometer, equipped with a PHOTON 100 CMOS detector and an INCOATEC microfocus source with Quazar mirror optics (Mo-Kα radiation, λ= 0.71073 Å). The data obtained were integrated with SAINT and a semi-empirical absorption correction from equivalents with SADABS was applied. The structure was solved and refined using the Bruker SHELX 2014 software package.[1] All non-hydrogen atoms were refined with anisotropic displacement parameters. All C-H hydrogen atoms were refined isotropically on calculated positions by using a riding model with their U_{iso} values constrained to 1.5 U_{eq} of their pivot atoms for terminal sp3 carbon atoms and 1.2 times for all other carbon atoms.

5.2. Crystallographic details of **2**^{L2}

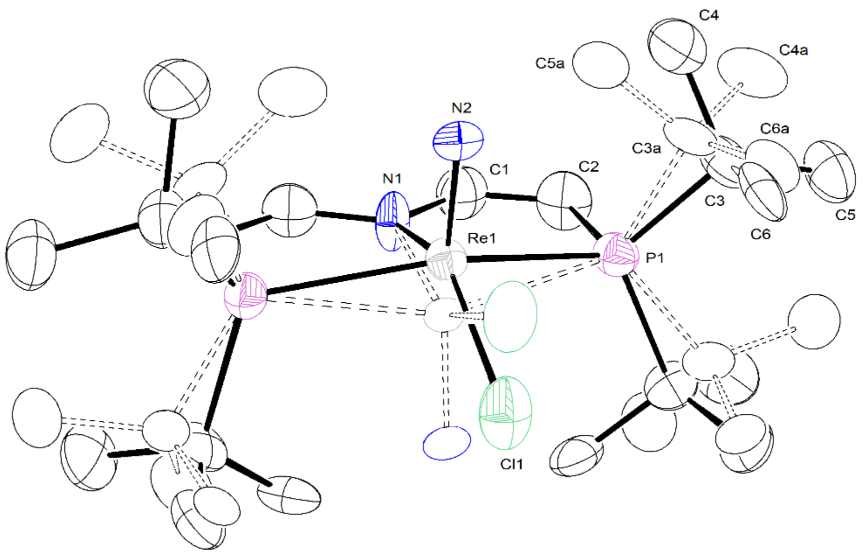

Figure S27. Thermal ellipsoid plot of **2**^{L2} with the anisotropic displacement parameters drawn at

the 50% probability level. The asymmetric unit contains a half disordered complex molecule. The disorder was refined with site occupation factors of 0.5 for both sites using PART commands and some restraints (RIGU). The structure was refined as an inversion twin using the twin law -100 0-10 00-1 (BASF: 0.50(3)).

Table S1. Crystal data and structure refinement for **2^{L2}**.

CCDC code	1832926	
Empirical formula	$C_{20}H_{40}ClN_2P_2Re$	
Formula weight	592.13	
Temperature	100(2) K	
Wavelength	0.71073 Å	
Crystal system	Tetragonal	
Space group	$P\text{-}42_1m$	
Unit cell dimensions	a = 12.0771(7) Å	□= 90°
	b = 12.0771(7) Å	□= 90°
	c = 8.3696(6) Å	□ = 90°
Volume	1220.76(17) Å^3	
Z	2	
Density (calculated)	1.611 Mg/m^3	
Absorption coefficient	5.225 mm^{-1}	
F(000)	592	
Crystal size	0.234 x 0.229 x 0.130 mm^3	
Crystal shape and color	Block,	light brown
Theta range for data collection	2.385 to 27.443°	
Index ranges	-15<=h<=15, -15<=k<=15, -10<=l<=10	
Reflections collected	20488	
Independent reflections	1492 [R(int) = 0.0883]	
Completeness to theta = 25.242°	100.0 %	
Max. and min. transmission	0.7457 and 0.5234	
Refinement method	Full-matrix least-squares on F^2	
Data / restraints / parameters	1492 / 36 / 121	
Goodness-of-fit on F^2	1.151	
Final R indices [I>2sigma(I)]	R1 = 0.0444,	wR2 = 0.0841
R indices (all data)	R1 = 0.0611,	wR2 = 0.0925
Absolute structure parameter	0.50(3)	
Largest diff. peak and hole	1.888 and -1.191 eÅ^{-3}	

Table S2. Bond lengths [Å] and angles [°] $\mathbf{2}^{L2}$.

Re(1)-Re(1)#1	0.7325(14)
Re(1)-N(2)	1.647(18)
Re(1)-N(1)	2.106(13)
Re(1)-Cl(1)#1	2.201(7)
Re(1)-N(2)#1	2.373(19)
Re(1)-Cl(1)	2.395(7)
Re(1)-P(1)#1	2.447(3)
Re(1)-P(1)	2.447(3)
Cl(1)-Cl(1)#1	1.220(18)
Cl(1)-Re(1)#1	2.201(7)
P(1)-C(2)	1.780(14)
P(1)-C(3)	1.86(3)
P(1)-C(3)#2	1.86(3)
P(1)-C(3A)#2	1.88(3)
P(1)-C(3A)	1.88(3)
P(1)-Re(1)#1	2.447(3)
N(1)-C(1)	1.354(17)
N(1)-C(1)#1	1.354(17)
N(1)-Re(1)#1	2.106(13)
N(2)-Re(1)#1	2.373(19)
C(1)-C(2)	1.35(2)
C(3)-C(4)	1.53(3)
C(3)-C(6)	1.54(5)
C(3)-C(5)	1.54(3)
C(3A)-C(6A)	1.50(6)
C(3A)-C(5A)	1.51(3)
C(3A)-C(4A)	1.53(4)

Re(1)#1-Re(1)-N(2)	170.8(5)
Re(1)#1-Re(1)-N(1)	79.98(7)
N(2)-Re(1)-N(1)	109.2(5)
Re(1)#1-Re(1)-Cl(1)#1	96.4(2)
N(2)-Re(1)-Cl(1)#1	74.4(6)
N(1)-Re(1)-Cl(1)#1	176.3(3)

Re(1)#1-Re(1)-N(2)#1	6.4(4)
N(2)-Re(1)-N(2)#1	164.4(9)
N(1)-Re(1)-N(2)#1	86.4(4)
Cl(1)#1-Re(1)-N(2)#1	90.0(4)
Re(1)#1-Re(1)-Cl(1)	66.0(2)
N(2)-Re(1)-Cl(1)	104.8(6)
N(1)-Re(1)-Cl(1)	145.9(2)
Cl(1)#1-Re(1)-Cl(1)	30.4(4)
N(2)#1-Re(1)-Cl(1)	59.6(4)
Re(1)#1-Re(1)-P(1)#1	81.39(2)
N(2)-Re(1)-P(1)#1	99.94(8)
N(1)-Re(1)-P(1)#1	79.71(8)
Cl(1)#1-Re(1)-P(1)#1	99.82(9)
N(2)#1-Re(1)-P(1)#1	82.44(7)
Cl(1)-Re(1)-P(1)#1	94.62(9)
Re(1)#1-Re(1)-P(1)	81.39(2)
N(2)-Re(1)-P(1)	99.94(8)
N(1)-Re(1)-P(1)	79.71(8)
Cl(1)#1-Re(1)-P(1)	99.82(9)
N(2)#1-Re(1)-P(1)	82.44(7)
Cl(1)-Re(1)-P(1)	94.62(9)
P(1)#1-Re(1)-P(1)	155.11(13)
Cl(1)#1-Cl(1)-Re(1)#1	83.6(2)
Cl(1)#1-Cl(1)-Re(1)	66.0(2)
Re(1)#1-Cl(1)-Re(1)	17.70(7)
C(2)-P(1)-C(3)	107.8(11)
C(2)-P(1)-C(3)#2	107.8(11)
C(3)-P(1)-C(3)#2	94.3(16)
C(2)-P(1)-C(3A)#2	104.6(12)
C(2)-P(1)-C(3A)	104.6(12)
C(3A)#2-P(1)-C(3A)	128.0(17)
C(2)-P(1)-Re(1)	99.0(6)
C(3)-P(1)-Re(1)	130.9(7)
C(3)#2-P(1)-Re(1)	115.8(7)
C(3A)#2-P(1)-Re(1)	100.2(8)
C(3A)-P(1)-Re(1)	116.5(8)

C(2)-P(1)-Re(1)#1	99.0(6)
C(3)-P(1)-Re(1)#1	115.8(7)
C(3)#2-P(1)-Re(1)#1	130.9(7)
C(3A)#2-P(1)-Re(1)#1	116.5(8)
C(3A)-P(1)-Re(1)#1	100.2(8)
Re(1)-P(1)-Re(1)#1	17.22(4)
C(1)-N(1)-C(1)#1	120.3(16)
C(1)-N(1)-Re(1)#1	119.3(8)
C(1)#1-N(1)-Re(1)#1	119.3(8)
C(1)-N(1)-Re(1)	119.3(8)
C(1)#1-N(1)-Re(1)	119.3(8)
Re(1)#1-N(1)-Re(1)	20.04(13)
Re(1)-N(2)-Re(1)#1	2.84(17)
C(2)-C(1)-N(1)	124.4(14)
C(1)-C(2)-P(1)	115.7(12)
C(4)-C(3)-C(6)	112(3)
C(4)-C(3)-C(5)	112(2)
C(6)-C(3)-C(5)	104(3)
C(4)-C(3)-P(1)	105.8(19)
C(6)-C(3)-P(1)	109(2)
C(5)-C(3)-P(1)	114.6(16)
C(6A)-C(3A)-C(5A)	106(4)
C(6A)-C(3A)-C(4A)	108(3)
C(5A)-C(3A)-C(4A)	111(3)
C(6A)-C(3A)-P(1)	115(3)
C(5A)-C(3A)-P(1)	104.8(16)
C(4A)-C(3A)-P(1)	113(2)

Symmetry transformations used to generate equivalent atoms:
#1 -x+1,-y,z #2 y+1/2,x-1/2,z

Table S3. Torsion angles [°] for $\mathbf{2}^{L2}$.

N(1)-Re(1)-N(2)-Re(1)#1	180.000(14)
Cl(1)#1-Re(1)-N(2)-Re(1)#1	0.000(14)
N(2)#1-Re(1)-N(2)-Re(1)#1	0.000(11)
Cl(1)-Re(1)-N(2)-Re(1)#1	0.000(14)

P(1)#1-Re(1)-N(2)-Re(1)#1	-97.52(15)
P(1)-Re(1)-N(2)-Re(1)#1	97.52(15)
C(1)#1-N(1)-C(1)-C(2)	180.000(1)
Re(1)#1-N(1)-C(1)-C(2)	11.51(15)
Re(1)-N(1)-C(1)-C(2)	-11.51(15)
N(1)-C(1)-C(2)-P(1)	0.000(1)
C(3)-P(1)-C(2)-C(1)	-129.6(7)
C(3)#2-P(1)-C(2)-C(1)	129.6(7)
C(3A)#2-P(1)-C(2)-C(1)	111.8(8)
C(3A)-P(1)-C(2)-C(1)	-111.8(8)
Re(1)-P(1)-C(2)-C(1)	8.72(3)
Re(1)#1-P(1)-C(2)-C(1)	-8.72(3)
C(2)-P(1)-C(3)-C(4)	46.8(19)
C(3)#2-P(1)-C(3)-C(4)	157.2(13)
Re(1)-P(1)-C(3)-C(4)	-73(2)
Re(1)#1-P(1)-C(3)-C(4)	-63(2)
C(2)-P(1)-C(3)-C(6)	167(3)
C(3)#2-P(1)-C(3)-C(6)	-83(3)
Re(1)-P(1)-C(3)-C(6)	47(3)
Re(1)#1-P(1)-C(3)-C(6)	57(3)
C(2)-P(1)-C(3)-C(5)	-77(2)
C(3)#2-P(1)-C(3)-C(5)	33(3)
Re(1)-P(1)-C(3)-C(5)	163.2(17)
Re(1)#1-P(1)-C(3)-C(5)	173(2)
C(2)-P(1)-C(3A)-C(6A)	-167(3)
C(3A)#2-P(1)-C(3A)-C(6A)	-44(5)
Re(1)-P(1)-C(3A)-C(6A)	85(3)
Re(1)#1-P(1)-C(3A)-C(6A)	91(3)
C(2)-P(1)-C(3A)-C(5A)	78(3)
C(3A)#2-P(1)-C(3A)-C(5A)	-159.9(13)
Re(1)-P(1)-C(3A)-C(5A)	-30(3)
Re(1)#1-P(1)-C(3A)-C(5A)	-24(3)
C(2)-P(1)-C(3A)-C(4A)	-43(2)
C(3A)#2-P(1)-C(3A)-C(4A)	80(4)
Re(1)-P(1)-C(3A)-C(4A)	-150.6(17)
Re(1)#1-P(1)-C(3A)-C(4A)	-145(2)

Symmetry transformations used to generate equivalent atoms:
#1 -x+1,-y,z #2 y+1/2,x-1/2,z

Table S4. Hydrogen bonds for **2**^{L2} [Å and °].

D-H...A	d(D-H)	d(H...A)	d(D...A)	<(DHA)
C(1)-H(1)...Cl(1)#3	0.95	2.80	3.681(17)	154.3

Symmetry transformations used to generate equivalent atoms:
#1 -x+1,-y,z #2 y+1/2,x-1/2,z #3 y+1/2,x-1/2,z+1

6 References

[1] a) APEX2 v2014.9-0 (SAINT/SADABS/SHELXT/SHELXL), Bruker AXS Inc., Madison, WI, USA, **2014**. b) G.M. Sheldrick. SHELXT *Acta Cryst.,* **2015**, A71, 3-8. c) G. M. Sheldrick *Acta Cryst*., **2015**, C71, 3-8. d) G. M. Sheldrick *Acta Cryst*., **2008**, A64, 112-122.
